# Influence of mirror therapy and motor imagery on intermanual transfer effects in upper-limb prosthesis training of healthy participants: A randomized pre-posttest study

**DOI:** 10.1371/journal.pone.0204839

**Published:** 2018-10-09

**Authors:** Sietske Romkema, Raoul M. Bongers, Corry K. van der Sluis

**Affiliations:** 1 University of Groningen, University Medical Center Groningen, Department of Rehabilitation Medicine, Groningen, the Netherlands; 2 University of Groningen, University Medical Center Groningen, Center of Human Movement Sciences, Groningen, the Netherlands; University of Exeter, UNITED KINGDOM

## Abstract

The effect that a motor skill trained on one side can lead to improvement in the untrained side is called intermanual transfer. Intermanual transfer can help enhance upper limb prosthetic training. To determine the influence of mirror therapy and motor imagery on intermanual transfer in upper limb prosthesis training, a pseudo-randomized clinical trial, single blinded, with a pre-posttest design was used. Forty-seven able-bodied, right-handed participants were pseudo-randomly assigned to two training groups and one control group. One training group undertook an intermanual transfer training program, using an upper-limb prosthetic simulator with added mirror therapy and motor imagery. The second training group completed only the intermanual transfer training program. The control group completed a sham training: a dummy training without using the prosthesis simulator. The program lasted five consecutive days. To determine the improvement in skill, a test was administered before, immediately after, and six days after the training program. Training used the “unaffected” arm; tests were performed with the “affected” arm, resembling the amputated limb. Movement time, the time from the beginning of the movement until completion of the task; hand opening, the duration of the maximum prosthetic hand opening; and grip-force control, the deviation from the required force during a tracking task. No intermanual transfer effects were found: neither the intermanual transfer training program, nor the additional mirror therapy and motor imagery affected prosthesis skills. A limitation of the study was that the training program was applied to able-bodied subjects instead of patients with an amputation. Contrary to previous studies, no intermanual transfer effects were found. Additional mirror therapy and motor imagery did not ameliorate intermanual transfer effects.

## Introduction

**Fig 1:**
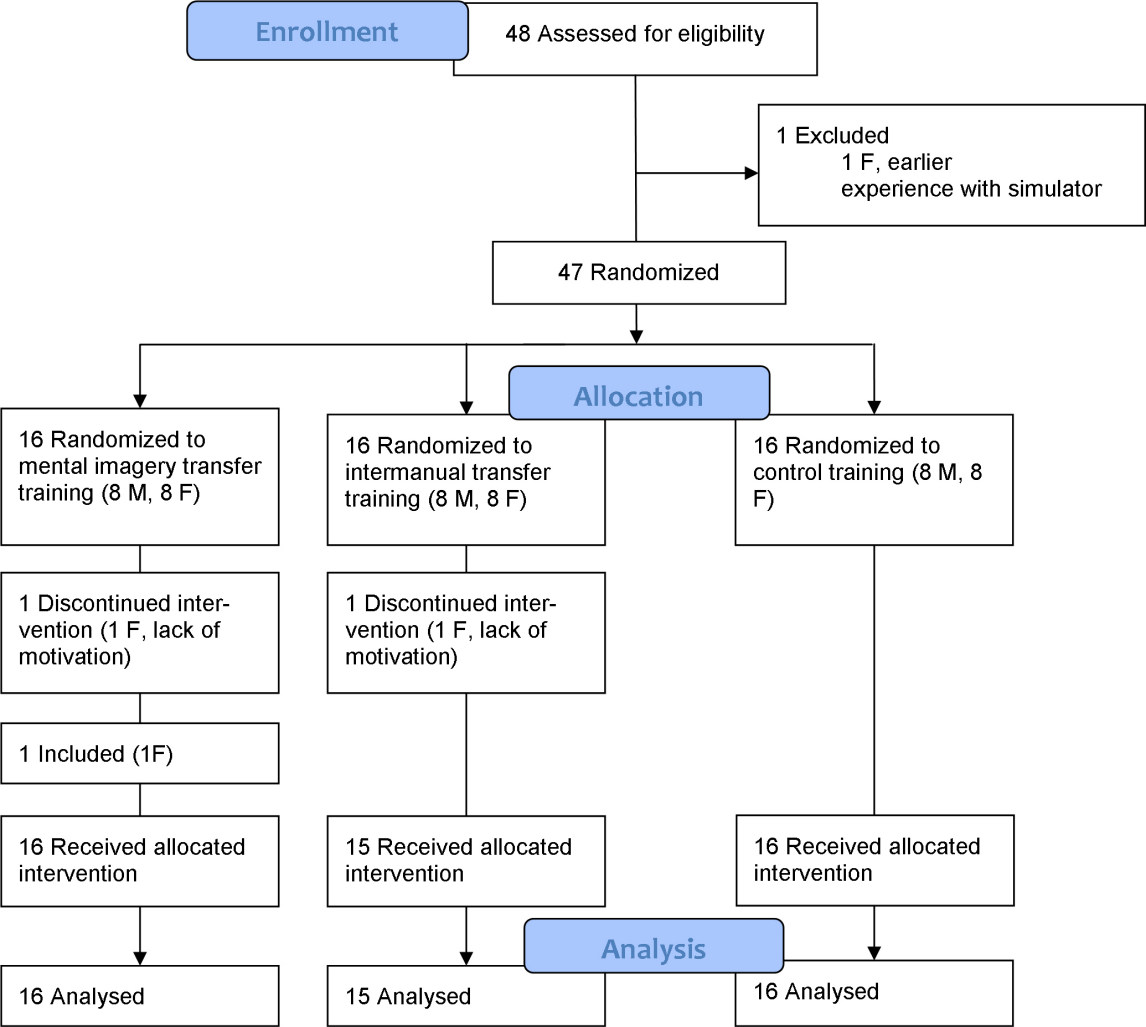
Consort flow diagram.

Intermanual transfer effects can be used to improve prosthesis training in able-bodied[1–4] and amputees [5]. Intermanual transfer involves motor skills trained on one side of the body transferring to the other side.[6–9] For prosthetic training this means that, when the unaffected hand is trained, motor skills of the affected limb improve. The rationale for using intermanual transfer in prosthetic training is that training can start early in rehabilitation and, as a result, might improve handling and acceptance of the prosthesis.[10–12] Even though the transfer effect has previously been shown in prosthetic training in able-bodied[1–4] as well as in persons with an amputation[5], the effect was found to be rather small. This study (Fig 1) aims to ameliorate the intermanual transfer effect in prosthetic training by additionally stimulating plasticity processes employing mirror therapy and motor imagery.

By using mirror therapy, an (upper) extremity is trained by watching the mirror reflection of the contralateral side. This generates a visual perception of movement execution with the extremity that is either missing[13] or, in our case, lying motionless behind the mirror. Although more research is necessary, mirror therapy seems useful for functional recovery and learning.[14,15] Motor imagery is the cognitive process of imagining a movement without actually executing it.[16–18] Many reviews,[19] discussing all kinds of mental practice interventions found beneficial effects for motor imagery, when used in combination with physical practice. The effectiveness of motor imagery is found to depend on intervention techniques, such as timing and task.[20–22] We assumed both mirror therapy and motor imagery could be beneficial for intermanual transfer. Cerebral motor area activity during motor imagery and mirror therapy resembles activity when actually executing a movement.[23–25] Although brain activation during intermanual transfer takes place in similar brain areas as when executing a movement,[26] the neural processes leading to the transfer effect are not fully understood.[27]

**Fig 2:**
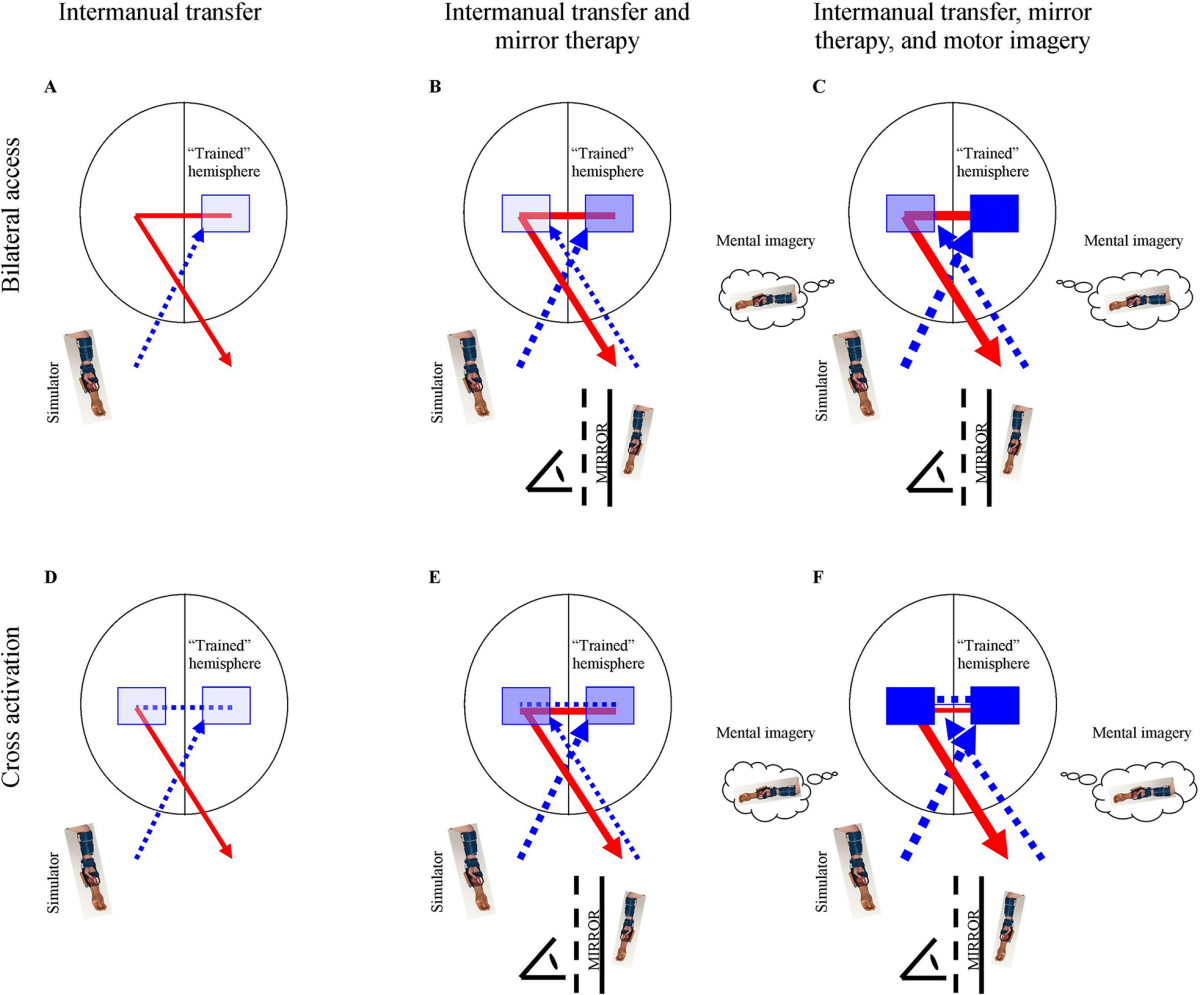
A schematic representation of the bilateral access and cross activation models for intermanual transfer training, extended using mirror therapy, and extended using motor imagery. The blue lines represent the input after training resulting in the activation, shown in blue squares. The shade of the squares reflects the degree of activation. The red lines represent the expected skill improvement due to the reflected brain activation. According to the bilateral access models, after training one side, a training effect should be established in the contralateral hemispheric motor cortex (A). After actual manual training (with the prosthesis simulator), the contralateral hemispheric motor cortex is activated. This “trained” hemisphere is accessible to control the untrained limb, resulting in intermanual transfer. By including mirror therapy (B), the “untrained” hemisphere will also be activated in the same motor cortex area, leading to increased transfer effects in the untrained limb. By extending the training with motor imagery, the hemispheric activation is expected to increase further (C). According to the cross activation models, after training one limb both hemispheres should be activated (D). The ipsilateral untrained hemisphere would thus also be activated. Note that this hemisphere is used when the untrained limb is performing manual practice. The supplemental mirror therapy (E) and motor imagery (F) are expected to increase the effect of intermanual transfer, whereby similar motor cortex areas are activated. The mirror therapy and motor imagery are supposed to have an effect on both sides.

The two main model types suggested in the literature to explain intermanual transfer–the bilateral access models and the cross activation models (Fig 2A and 2D)[28]–may help in understanding how these interventions might add to intermanual transfer. Focusing first on the bilateral access models, the hemispheric motor cortex contralateral to the trained side (for clarity called the “trained” hemisphere) is activated during motor skill learning. When the other untrained hemisphere is used, access to the trained hemisphere is assumed to be possible, which may explain the existence of intermanual transfer effects. By making use of this “trained” hemisphere the motor skills of the “untrained” side improve. Adding a stimulation training program (mirror therapy and motor imagery) may enhance the intermanual transfer effects.

In this bilateral access model, adding mirror therapy to intermanual transfer training implies that not only the contralateral hemisphere but also the ipsilateral, “untrained” hemisphere (i.e., controlling the affected arm) is trained (Fig 2B and 2C).[29] Additional mirror therapy may therefore be expected to lead to even better performance of the untrained side. Furthermore, with mirror therapy visual feedback from the untrained side is added, this should further help to activate this “untrained” hemisphere.[30] With motor imagery, there is a chance that the transfer effect might be even greater. Motor imagery of one extremity is understood to lead to bilateral activation of relevant brain areas.[31,32] This assumption is supported by the finding that the effects of mental practice can transfer to the untrained side.[27,33,34] To ensure that both hemispheres are activated, imagining motor skills can occur for both limbs so that this imagery training will further activate the relevant hemispheric motor cortex. This should lead to skill improvement by bolstering the effect of the intermanual transfer.

According to the second type of model (cross activation), both hemispheres are directly activated during motor learning (Fig 2D). When executing the motor skill with the untrained limb, the contralateral hemisphere–already improved by training the other limb–is also used. By adding mirror therapy and motor imagery to the intermanual transfer training program, both hemispheres undergo additional practice (Fig 2E and 2F). Furthermore, visual feedback from the untrained side extends the training program. Therefore, this model also supports the hypothesis that mirror therapy and motor imagery can strengthen intermanual transfer effects. Note that the current study is not designed to examine differences in the explanatory value of the bilateral access model and the cross activation model; these models are presented to provide a basis for how mirror therapy and motor imagery may strengthen intermanual transfer.

Earlier studies testing the effects of mirror therapy and intermanual transfer compared both types of training programs. The effect of mirror therapy has been found to be greater than the effect of intermanual transfer training.[30,35]

A few additional articles focused on both intermanual transfer and motor imagery.[27,33,34,36] Two studies[27,34] compared the transfer effects of actual manual training to mental practice of movements. The results favored mental practice. Another study[33] compared physical training, mental practice, and a combination of both, and found that alternating physical and mental practice had the greatest effect. Note that the above-mentioned mental practice studies applied intermanual transfer and mental practice training to the same single side. Although limited evidence shows that motor imagery of both sides has similar effects on the performance of one of them,[36] in our study we combined intermanual transfer training on one side with motor imagery training for both sides. When applying motor imagery to patients with an amputation, it might be helpful to add a motor imagery training program to the affected “untrained” side in order to activate the contralateral, “untrained” hemisphere and facilitate direct access.

Using a prosthesis was a new skill for the prosthesis simulator users. We assumed that this new skill could be learned using motor imagery, because the necessary movements for operating a myoelectric prosthesis (wrist extension and flexion) were familiar. However, we were cautious about this assumption, because an earlier study showed that a novel movement could not be learned through motor imagery.[37] To make sure that the movement was not entirely novel, we started our training with physical practice (of the unaffected side) using a prosthesis simulator (a prosthesis that can be worn on a sound hand) and mirror therapy, before using motor imagery.

The aim of the current study was to reveal whether the combination of motor imagery and mirror therapy helped improve intermanual transfer training with a prosthesis simulator. By comparing a mirror therapy and motor imagery training, an intermanual transfer training and a sham training, the presence of an intermanual transfer effect and the effect of the additional therapies was tested. Based on literature and theoretical models, the intermanual transfer effect was expected to be present and to improve with the additional therapies.

## Methods

### Design overview

**Fig 3:**
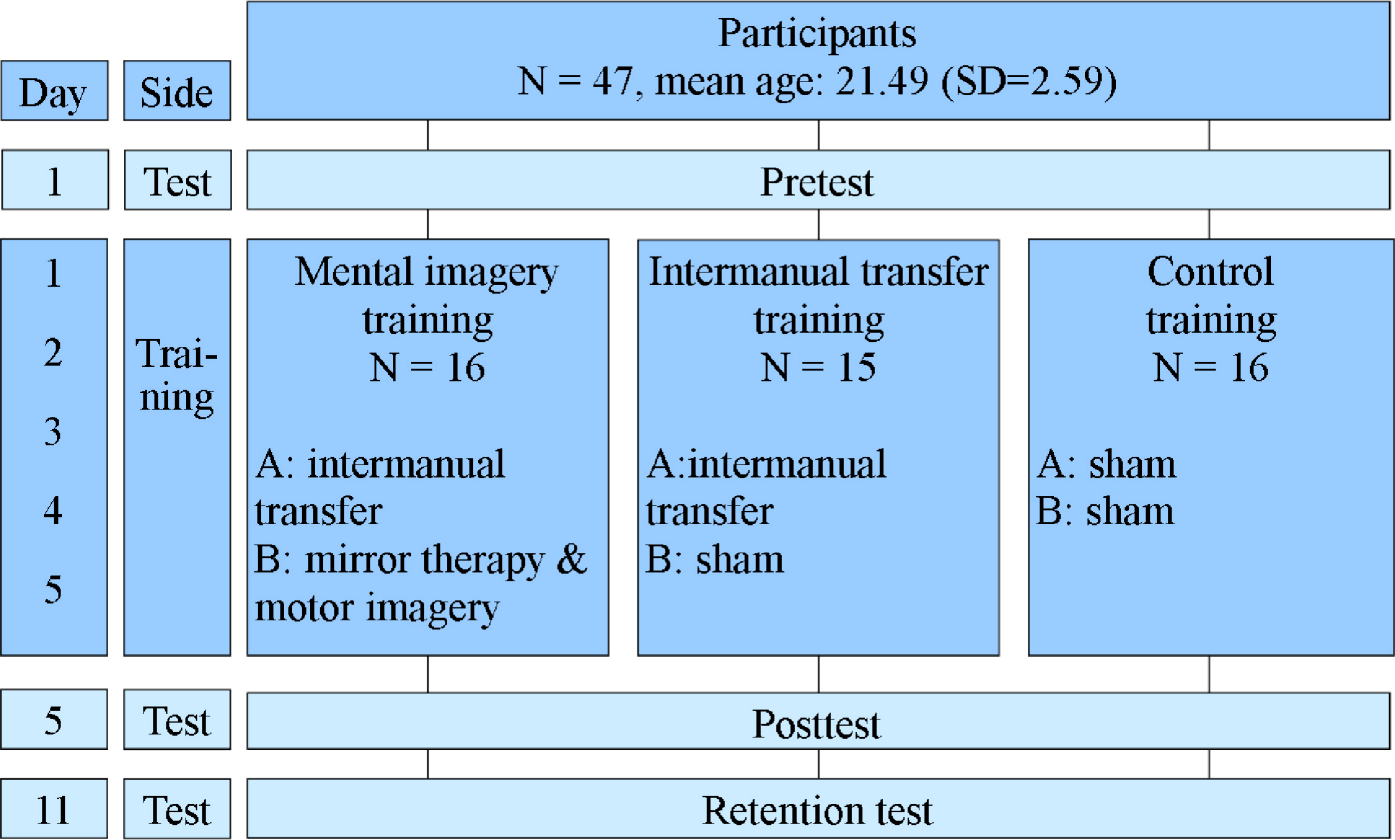
The design of the experiment. Each training session was split into two parts (A and B).

The consort checklist was used to report the randomized trial (S1 Consort checklist). Participants were, according to the trial study protocol (S1 File), divided into three groups (Fig 3). One group received intermanual transfer training and mirror therapy and motor imagery training (IT-MTMI group); the second group received only intermanual transfer training (IT group). Both groups used the prosthetic simulator in the training programs. The third group was a control group (CO group) that received a sham training, a dummy training without the prosthesis simulator.

All groups started with a pretest (day 1) to establish the prosthesis skills of the participants’ “affected” arm with the simulator. They then practiced for five consecutive days with the opposite “unaffected” arm. Subsequently, participants performed a posttest and six days later a retention test, using the simulator on the “affected” arm. The retention test was meant to investigate whether the learned skills remained. The training sessions as well as the test sessions comprised functional and grip-force control tasks.

### Setting and participants

Forty-seven right-handed, able-bodied volunteers were recruited and measured between April and July 2014. The sample size calculation was based on force control data from an experiment where participants had also trained five days with the prosthesis simulator.[38] Using G*Power,[39] we estimated the number of participants to be 14 per group in order to reach a power of 0.8 between the intermanual transfer and sham group. The effect size was .82; a type I error of 0.05 and an a priori, double-sided t-test based on differences between two independent means were used. Controlling for an equal distribution of gender and test hand per group we included 16 participants per group. All participants signed an informed consent document in advance. After completion of the experiment, participants received a gift voucher. The training procedures and the consent form of this study were approved by the local medical ethics committee, the Medical Ethics Committee of the University Medical Center Groningen (METc UMCG, NL48028.042.14). The trial was registered with the Nederlands Trial Register (trialregister.nl, NTR4432).

All participants had no known neurologic or upper extremity musculoskeletal problems, had normal or corrected-to-normal sight, and had no earlier experience with the prosthesis simulator. Hand dominance was determined by the Edinburgh Handedness Inventory (EHI).[40]

The materials and procedures used in this study vis-à-vis the simulator, the fitting of the simulator, the time registration, and the force registration were similar to those we used in previous studies.[2,4,38]

**Fig 4:**
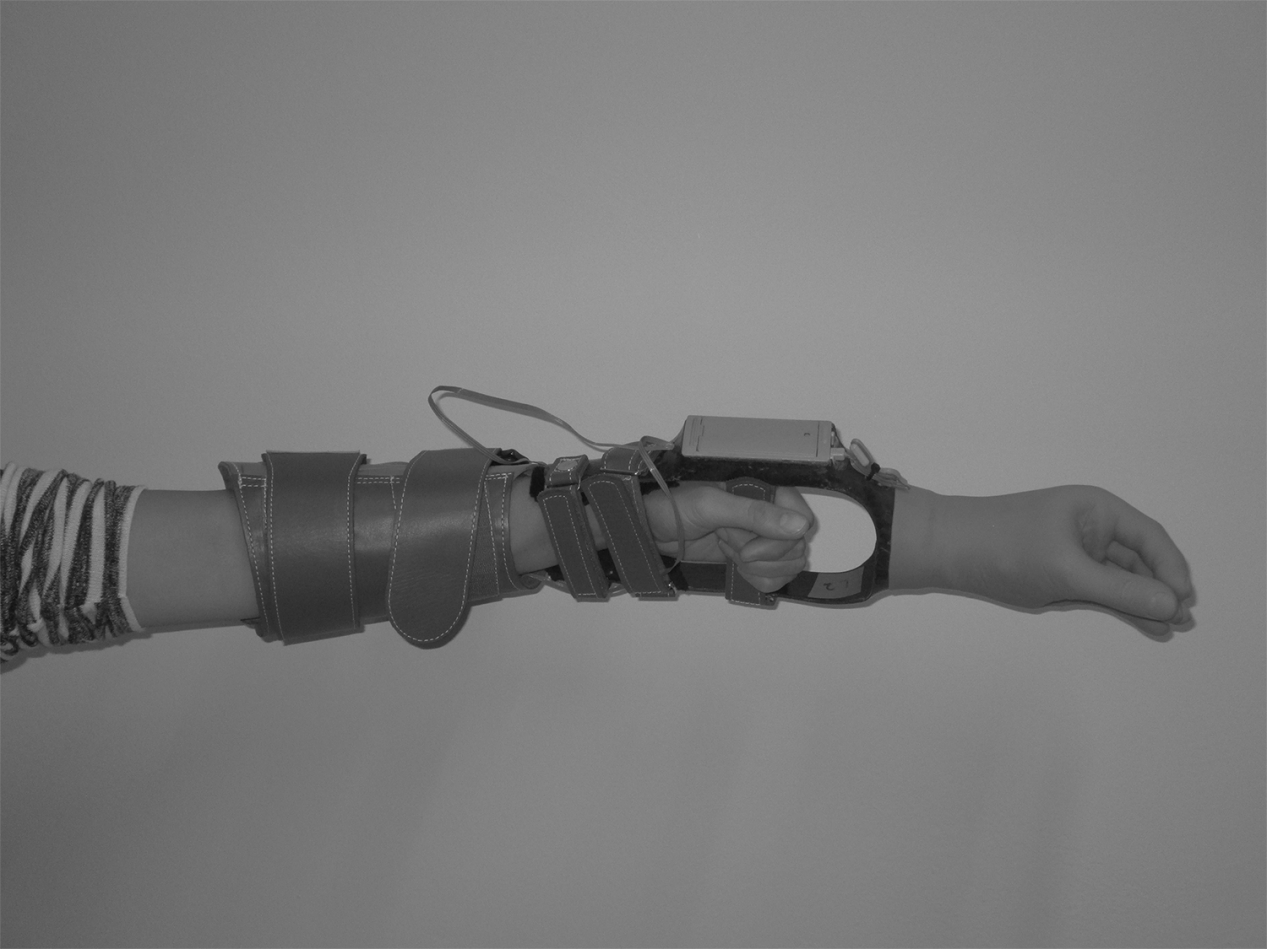
The prosthesis simulator.

The myoelectric prosthesis simulator (OIM Orthopedie, Haren, the Netherlands)[41,42] consisted of a myoelectric hand, the MyoHand VariPlus Speed (Otto Bock, Duderstadt, Germany), attached to an open cast in which the hand was placed (Fig 4). The cast extended into a splint along the forearm. The splint could be attached to the upper limb with a Velcro sleeve. The hand was controlled by changes in electrical activity related to muscle contraction, detected by two electrodes placed on the muscle bellies of the forearm. The maximum speed of the prosthesis hand was set to the default setting of six (dual site control). The prosthetic hand had proportional speed control (15–300 mm/s) and proportional grip-force control (0-±100 N).

To determine the correct sensitivity of the electrodes the MyoBoy (757M11 Myoboy; 13E200 MyoBock Electrodes; Otto Bock, Duderstadt, Germany) was used.

E-Prime (Psychology Software Distribution, York, UK) was used to measure movement time of the functional tasks, recorded in milliseconds. Before each trial, a computer screen to the left of the participant showed which task had to be executed. A keyboard was positioned alongside the arm being tested. At the start and at the end of the task, the participant pressed the spacebar to start and stop the time.

A potentiometer was used to measure the hand opening. Two rods were attached to the potentiometer. One was fixed to the thumb and one to the index finger of the prosthesis hand. The output of the potentiometer indicated the angle between the thumb and index finger. This output was digitally sampled with a 32-channel Porti system (TMSI, Enschede, the Netherlands).

**Fig 5:**
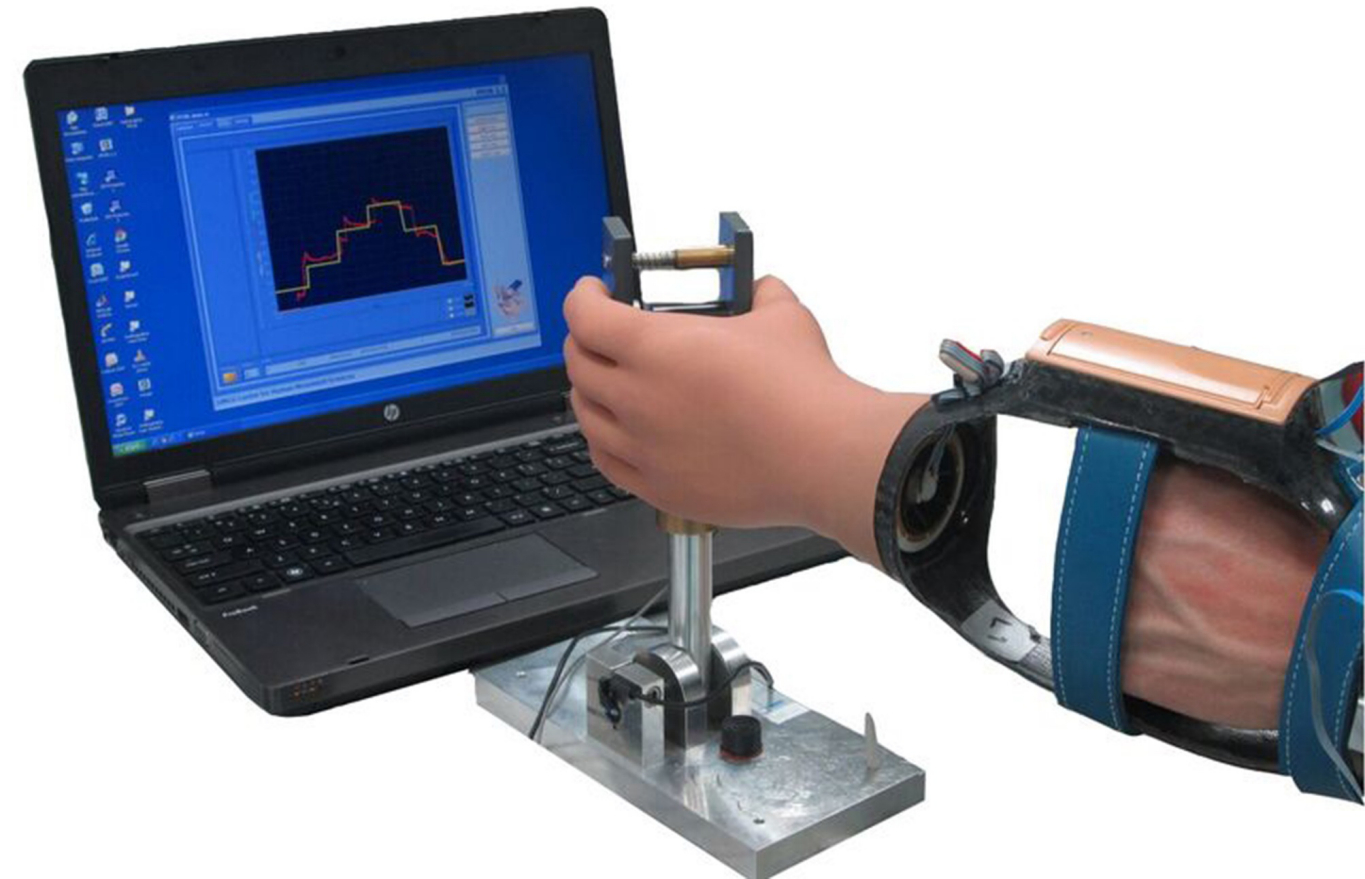
Force control training using the prosthesis simulator and a custom-made tracking program controlled by a handle.

In order to measure the force when pinching a handle (Fig 5), a custom-made program, created with Labview (display and sample frequency 100 Hz), was used.[43] The handle consisted of two plates (6 x 3.5 x 9 cm), with a force transducer (LLB350 Loadcell [Futek]) placed in between measuring the force. The task consisted of tracking a line shown on the screen, with the actual forces also shown on the screen.[4]

### Randomization and interventions

The participants were pseudo-randomly divided over the three groups by using a computer-generated random-number sequence. Men and women were equally divided over the three groups. The assessors were blinded for the training program the participant had followed. It was physically impossible to blind participants because they were aware of training with or without the prosthesis simulator.

All test and training sessions using the prosthesis simulator started with a standard procedure to fit the simulator. Electrodes were placed on the wrist muscles. To set the electrode sensitivity, the amplified signal on the MyoBoy had to exceed a threshold of 1.5 V (high signal) sustained for two seconds. A maximum of five contractions was allowed to minimize training effects. After the simulator was fitted, the participant was positioned in front of the table, with the elbow flexed 90 degrees. Verbal instruction was given on task execution.

#### Pretest, posttest, and retention test

The pretest, posttest, and retention test consisted of three functional tasks and one grip-force control task. All tasks were performed three times in blocked random order, lasting no more than 15 minutes. Participants were instructed to execute all tasks as rapidly and accurately as possible. The functional tasks were based on findings of Van Lunteren and were related to the different ways prostheses are used in daily life.[44] The tasks were the mug task (direct grasping), jar-lid task (indirect grasping) and pen-case task (fixating), as used in earlier studies.[2–4,42] During the mug task, the duration of the maximum hand opening was recorded, using a Labview custom-made program. The grip-force control task was a tracking task, where a line was shown on the screen. By controlling the force on the handle the pattern on the screen could be followed.[4]

#### Training sessions

All groups executed a 45-minute training program for five days, with (IT-MTMI and IT groups) or without (CO-group) the prosthetic simulator. All training sessions were split into two parts: part A lasting 30 minutes and part B lasting 15 minutes.

#### Mirror therapy and motor imagery training

Before the training started the IT-MTMI group filled in the Vividness of Movement Imagery Questionnaire[45], to assess their ability to imagine. For the IT-MTMI group, part A was divided into 20 minutes of functional training and 10 minutes of grip-force control training, given in random order. For the functional part, participants trained using the tasks from SHAP.[46] SHAP consists of 26 tasks: twelve abstract-object tasks and 14 activities-of-daily-life tasks, all to be performed with the prosthetic hand. During the grip-force control part, participants practiced different patterns of the tracking task, offered in random order. Part B consisted of 15 minutes, ten minutes dedicated to mirror therapy and five on motor imagery. During the mirror therapy the prosthesis simulator was worn and put in front of a mirror, which was positioned in the sagittal plane of the participant. The arm wearing a prosthesis simulator was seen in the mirror, creating the illusion that the arm hidden behind the mirror was wearing a prosthesis simulator. The arm actually wearing the simulator was covered with a box that was open on the mirror side so that the participant focused solely on its reflection. The training consisted of watching solely the reflection of the hand, moving the hand in space, opening and closing the hand at different speeds, and picking up (deformable) objects that were positioned right before the prosthetic hand. During the motor imagery training the participant sat, eyes closed with both hands positioned on a table. During this training the prosthesis simulator was not worn because we could not use it on the test hand, where we finally intended to measure the combined effect of intermanual transfer, mirror therapy and motor imagery. The participant was asked to imagine wearing a prosthetic simulator on the “unaffected” training hand (that previously wore the prosthesis simulator) while imagine executeing different movements. The participant then had to imagine the movements with the “affected” test hand that imaginary was wearing the prosthesis simulator. Hereby the participant was asked to imagine how the weight and length of the prosthesis would affect the effort to perform movements with the prosthesis and to activate the hand opening and closing. The requested imaginary movements consisted of the same tasks as used in mirror therapy training, including some bimanual tasks, such as opening a jar lid.

#### Intermanual transfer training

Part A for the IT-group consisted of the same functional training and grip-force control training as in the IT-MTMI group. However, part B contained a sham training, in which the participants were asked to play the card game Solitaire. No simulator was involved in this part of the training.

#### Sham training

The CO group underwent a sham training procedure, with no prosthetic simulator used. It comprised two parts, executed in random order. During part A, participants were asked to fill in sudokus for 30 minutes. During the 15 minutes of part B, like the IT group, they played the card game Solitaire. The participants were allowed to use their preferred hand.

### Outcomes

The primary outcome measures were 1) the movement time, the time in milliseconds from the beginning of the task until completion of the task; and 2) the duration of the maximum hand opening in milliseconds, while picking up an object (the time the hand is maximally opened).[47,48] The duration of the maximum hand opening was derived from the measurement with the potentiometer using Matlab (version 7.4). Finally, 3) the grip-force control, the difference between the required and the actual force, was produced in N (i.e., the deviation).

### Statistical analyses

Analyses were performed using Social Package Statistical Science (SPSS) 22.0 software package (SPSS, IBM Corp., Armonk, NY, USA). The means of the movement times, duration of maximum hand opening, and deviation in grip-force for the three trials in each test were calculated. To be able to compare the movement times of the different functional tasks, z-scores were calculated for the three test tasks. All outliers that deviated more than three times the standard deviation per test were removed at the level of the trial. Missing values were replaced using the expectation maximization algorithm in SPSS.

The three dependent variables (movement time, duration of the maximum hand opening, and deviation of the grip-force control) were analyzed with an ANCOVA. The data from the pretest were used as a covariate to be able to take into account possible differences between groups at baseline.

An ANCOVA was calculated for the *movement times* with test (posttest, retention test) and task (mug, jar lid, and pen case) as within-subject factors, and training group (IT-MTMI, IT, CO) and hand dominance (dominant and non-dominant) as between-subject factors. All three tasks of the pretests were used as covariates.

Two separate ANCOVAs were performed on the *duration of maximum hand opening* and *deviation of grip-force*, with test (posttest, retention test) as a within-subject factor, and training group (IT-MTMI, IT, CO) and hand dominance (dominant and non-dominant) as between-subject factors, in order to examine whether the groups were different. Again all three tasks of the pretests were used as covariates.

When sphericity was violated, the degrees of freedom were adjusted with the Greenhouse-Geisser correction. A significance criterion of 0.05 was used during the analysis. Post-hoc tests used a Bonferroni correction. For the significant effects, an effect size was calculated ŋ^2^_p_ and interpreted according to Cohen’s recommendation[49] of 0.02 for a small effect, 0.06 for a medium effect, and 0.14 for a large effect. Only effects with an effect size greater than 0.02 were reported in the results.

Forty-eight participants were eligible for the study, one of whom had to be excluded because she had previous experience with the prosthesis simulator (Fig 2). Two more females dropped out due to lack of motivation; one was replaced by another female. The participants (24 males, 23 females; mean age 21.49 years [SD = 2.59]) had a mean laterality quotient of 84 (range 50–100) on the EHI, meaning that all participants were right-handed. The Vividness of Movement Imagery Questionnaire showed that overall participants of the IT-MIMT group were able to imagine. Scores were between 48 and 158, while only two participants had a score higher than the mean number of points that could be gained, implicating that all other participants reported a clear and reasonably vivid imagery. The percentage of outliers that were removed was 2% for the movement time data, 4% for the maximum hand opening data, and 1% for the grip force control data.

## Results

### Movement time

No significant effect of the training program was found on movement times (Table 1). We did find a significant interaction effect for test, task, and training (F_4,58_ = 3.382, P = .015, ŋ^2^_p_ = .189). The MI group seemed to be faster on the posttest; we therefore performed two univariate ANOVAs comparing the training groups on the posttest and on the retention test, though no significant differences were found. The ANCOVA did show a main effect for the jar-lid task in the pretest (F_1,29_ = 4.454, P = .044, ŋ^2^_p_ = .133), showing that fast performance on the jar-lid task predicted fast movement times in the posttest and retention test.

**Table 1:** Means (95% Confidence Interval) for the movement times, duration of the maximum hand opening, and the deviation in grip-force control for the three groups per test.

Variable	Test	Motor imagery group	Intermanual transfer Group	Sham group
Movement time (ms)	Pretest	7536 (6977–8095)	7923 (7327–8518)	7348 (6755–7940)
	Posttest	4799 (4592–5005)	5286 (4897–5674)	5433 (5032–5834)
	Retention test	4612 (4341–4882)	4650(4387–4913.)	4718(4445–4990)
Duration of hand opening (ms)	Pretest	1093(921–1266)	1294 (1010–1578)	1143 (910–1378)
	Posttest	720 (604–837)	613 (394–832)	752 (622–883)
	Retention test	592 (460–725)	642 (509–775)	655(513–796)
Grip-force control (N)	Pretest	9.14 (6.62–11.65)	9.18 (7.16–11.21)	10.35 (8.36–12.33)
	Posttest	5.43 (4.64–6.21)	6.89 (5.47–8.31)	8.11 (6.17–10.04)
	Retention test	6.01 (4.78–7.24)	5.48 (4.71–6.25)	6.49 (5.15–7.83)

Note that, for the functional tasks, the real movement times are shown, while the analyses were performed on the z-scores.

### Duration of the maximum hand opening

The type of training did not show significant effects on the duration of the maximum hand opening. The only significant main effect was found for the pretest covariate (F_1,37_ = 6.134, P = .018, ŋ^2^_p_ = .142) which shows that a short duration of maximum hand opening in the pretest relates to a short duration in the posttest and retention test.

### Grip-force control

No significant effects were found for the deviation in grip-force control.

## Discussion

This was the first study where intermanual transfer training was extended, using a combination of mirror therapy and motor imagery training. An intermanual transfer, mirror therapy, and motor imagery training group was compared with a group training only intermanual transfer, and with a control group. Contrary to our expectations, no significant differences were found between the three training groups.

Several previous studies have shown intermanual transfer effects on movement times in prosthetic training.[1–4] Like the current study, these studies were executed by able-bodied subjects using a prosthesis simulator, and although the intermanual transfer effects were not large, they seemed robust. Recently, the intermanual transfer effect was also demonstrated in persons with an amputation[5]. The effect of intermanual transfer training was not found in the present study, but we did find an interaction effect for test, task, and training group. However, additional analyses did not show a clear influence of training group on this interaction.

Intermanual transfer of grip-force control could be expected based on the literature focusing on lifting with sound hands.[50,51] However, the fact that we did not find any effect on grip-force control was consistent with our previous work in prosthetic training on able-bodied subjects,[2–4] where no transfer effect for force control was found. If mirror therapy and motor imagery did influence the intermanual transfer effect, we would expect to find an effect on grip-force control, because a grip-force control task would benefit most from additional stimulation. A possible reason for not finding any effect on grip-force control is that grip-force control with a prosthesis is known to be hard to learn,[47,48,52] and possibly the intermanual transfer as well as the mirror therapy and motor imagery training were not focused specifically on grip-force control.

By adding mirror therapy and motor imagery to the intermanual transfer training we expected–based on the two main types of models explaining intermanual transfer–to be able to further stimulate existing brain activation with intermanual transfer training. The literature shows that both mirror therapy[14,15] and motor imagery[19] are expected to improve learning, and that the activated brain areas in mirror therapy, motor imagery, and intermanual transfer are similar to those used in actual performance.[23–26] Due to the presumed additional activation of the relevant brain regions, we expected to find an increase in the effect the training had. The absence of this increase might be due to the task features and the duration of the training programs. Although it is generally assumed that functional tasks are the most motivating and therefore the most successful, the tasks we used might have been too difficult and the training programs might have been too short. In the study of Crajé et al.,[53] an improvement in reaching and grasping was found after three weeks of daily motor imagery training, but no improvement was found in the more complex fine dexterity. The authors suggested that more complex tasks might need longer intervention periods.

The training tasks were molded in such a way that the effect would be maximal. The position of the subject was congruent to the task that was imagined, the imagination was combined with the physical practice and the functional tasks were imagined with an internal kinaesthetic perspective.[54] Though regarding the duration or frequency of the intervention, a review showed that studies on mirror therapy–although often with smaller groups–applied this intervention for three to eight weeks,[15] instead of the five days we did. Focusing on motor imagery, another literature review showed that apparently, although motor imagery sessions should be no longer than 20 minutes,[55] the mean number of training sessions which led to a positive result was 13,[19] instead of the five training sessions we used. Thus, even though we used the therapy as complementary–and it is unknown how many training sessions are needed to result in an effect under these circumstances–it is possible that the influence of mirror therapy and motor imagery would have been present had we used more training sessions or if the sessions had been distributed over a longer period.

In the introduction, two types of theoretical models were presented to explain intermanual transfer:[28] the bilateral access models and the cross-activation models (see Fig 1). Establishing which type is applicable in intermanual transfer was beyond the scope of the current study, making it impossible to determine the model used in intermanual transfer. The exact influence of mirror therapy and motor imagery on brain activation, and thus how the findings can be explained, also remains unknown. It would be interesting to study further which hemisphere is involved in which part of the process in order to understand how intermanual transfer works and how it can be improved by, among other things, mirror therapy and motor imagery training. This might help reveal ways to optimize intermanual transfer effects and which (combination of) therapies might have the most optimal effect on prosthesis training.

Although we tried to resemble a rehabilitation setting as much as possible, a limitation of this study is that we used able-bodied subjects instead of persons with an upper-limb amputation. The limited number of recent amputees means it is not feasible to reach the requirements of the sample size calculation. We assume that studies involving able-bodied participants allow general statements to be made about prosthesis use, because amputees generally are healthy and because the kinematic performance observed in simulators is comparable to performance with real prosthetic devices.[47]

Because of the null results the question raises if we have included a sufficient number of participants to show the correct results, despite the fact that we included the requested number of participants, based on the power calculation. This question should be answered in future research. In future research the possible familiarisation effects of the pre-test, which may explain the improvements from pre- to post-test in the sham group, should also get attention. Such a familiarisation effect could be made visible by applying more than one pre-test.

And although the positioning of the simulator is different than the positioning of the real prosthesis, we assume that this does not influence the magnitude of the effects of intermanual transfer for amputees, foremost because prosthetic skills of prosthesis wearers do transfer to the non-affected side operating a simulator [5]. Moreover, perceptual effects due to real and illusory weight differences were similar for prosthesis wearers and able-bodied participants wearing a simulator. In addition, the gaze behavior [56] of amputees and able-bodied subjects wearing a prosthesis simulator is comparable while other studies have shown that there are marked differences in gaze behavior between using the intact anatomical hand versus prosthesis users wearing a prosthesis [47] or able-bodied persons using a simulator [57]. Nevertheless, the possible effects of a limb amputation on the cortex, reflected in cerebral reorganization, are not present in able-bodied subjects and may bias the application of our results to prosthesis users.

Contrary to earlier findings, we did not find any intermanual transfer effects after prosthesis training. Furthermore, no training effects from mirror therapy and motor imagery were found. Further research is necessary to identify whether different tasks, more training sessions, or a longer period of training would lead to clinically relevant intermanual transfer effects in upper-limb amputees.

## Supporting information

S1 CONSORT Checklist CONSORT 2010 Checklist. (DOC)

S1 File Trial study protocol. (DOC)

## Abbreviations

ANOVA: Analysis of Variance
SHAP: Southampton Hand Assessment Procedure
SPSS: Social Package Statistical Science
