# Supplementary material for: Influence of mirror therapy and motor imagery on intermanual transfer effects in upper-limb prosthesis training of healthy participants: A randomized pre-posttest study
Source: PLoS One. 2018 Oct 9;13(10):e0204839. doi: 10.1371/journal.pone.0204839 (PMC6177130; doi:10.1371/journal.pone.0204839)
Supplement: S1 File — (DOC) [file pone.0204839.s002.doc]

**RESEARCH PROTOCOL**

Intermanual transfer using a mental imagery program in prosthetic training

**PROTOCOL TITLE** ‘Intermanual transfer using a mental imagery program in prosthetic training’

| **Protocol ID** | **48028** | |
| --- | --- | --- |
| **Short title** | **Intermanual transfer using mental imagery** | |
| **Version** | **2** | |
| **Date** | **28-03-2014** | |
| **Coordinating investigator/project leader** | **Raoul M. Bongers, MSc, PhD**  **Center for Human Movement Sciences,**  **University of Groningen,**  **UMCG, sector F**  **P.O. Box 196**  **9700 AD Groningen**  **(050) 363 8867**  **R.M.Bongers@umcg .nl** | **Prof. Corry. K. van der Sluis, MD, PhD**  **Center for Rehabilitation**  **UMCG**  **P.O. Box 30.001**  **9700 RB Groningen**  **(050) 361 2295**  **C.K.van.der.Sluis@umcg.nl** |
| **Principal investigator(s) (in Dutch: hoofdonderzoeker/uitvoerder)**  ***Multicenter research: per site*** | **Sietske Romkema, MSc**  **Centre for Rehabilitation**  **UMCG**  **P.O. Box 30.001**  **9700 RB Groningen**  **(050) 361 0108**  [**S.Romkema@umcg.nl**](mailto:S.Romkema@umcg.nl) | |
| **Sponsor (in Dutch: verrichter/opdrachtgever)** | ***Prof. Dr. C.K. van der Sluis*** | |
| **Subsidising party** |  | |
| **Independent expert** | **Rienk Dekker, MD, PhD**  **Centre for Rehabilitation**  **UMCG**  **P.O. Box 30.001**  **9700 RB Groningen**  **050 – 361 3638**  **R.Dekker@umcg.nl** | |
| **Laboratory sites <*if applicable*>** | **Not applicable** | |
| **Pharmacy <*if applicable*>** | **Not applicable** | |

**PROTOCOL SIGNATURE SHEET**

| **Name** | **Signature** | **Date** |
| --- | --- | --- |
| **Sponsor or legal representative:**  ***The research is non-commercial***  **For non-commercial research,**  **Head of Department:**  **Prof. dr. J.H.B Geertzen** |  |  |
| **Coordinating Investigator/Project leader/Principal Investigator:**  **Prof. Dr. C.K. van der Sluis** |  |  |

**TABLE OF CONTENTS**

1. INTRODUCTION AND RATIONALE [7](#__RefHeading___Toc354490671)

2. OBJECTIVES [10](#__RefHeading___Toc354490672)

3. STUDY DESIGN [10](#__RefHeading___Toc354490673)

4. STUDY POPULATION [13](#__RefHeading___Toc354490674)

4.1 Population (base) [13](#__RefHeading___Toc354490675)

4.2 Inclusion criteria [13](#__RefHeading___Toc354490676)

4.3 Exclusion criteria [13](#__RefHeading___Toc354490677)

4.4 Sample size calculation [13](#__RefHeading___Toc354490678)

5. TREATMENT OF SUBJECTS [14](#__RefHeading___Toc354490679)

5.1 Investigational product/treatment [14](#__RefHeading___Toc354490680)

5.2 Use of co-intervention (if applicable) [14](#__RefHeading___Toc354490681)

5.3 Escape medication (if applicable) [14](#__RefHeading___Toc354490682)

6. INVESTIGATIONAL PRODUCT [14](#__RefHeading___Toc354490683)

7. NON-INVESTIGATIONAL PRODUCT [14](#__RefHeading___Toc354490684)

8. METHODS [14](#__RefHeading___Toc354490685)

8.1 Study parameters/endpoints [14](#__RefHeading___Toc354490686)

8.1.1 Main study parameter/endpoint [14](#__RefHeading___Toc354490687)

8.1.2 Secondary study parameters/endpoints (if applicable) [14](#__RefHeading___Toc354490688)

8.1.3 Other study parameters (if applicable) [14](#__RefHeading___Toc354490689)

8.2 Randomisation, blinding and treatment allocation [14](#__RefHeading___Toc354490690)

8.3 Study procedures [14](#__RefHeading___Toc354490691)

8.4 Withdrawal of individual subjects [18](#__RefHeading___Toc354490692)

8.4.1 Specific criteria for withdrawal (if applicable) [18](#__RefHeading___Toc354490693)

8.5 Replacement of individual subjects after withdrawal [18](#__RefHeading___Toc354490694)

8.6 Follow-up of subjects withdrawn from treatment [18](#__RefHeading___Toc354490695)

8.7 Premature termination of the study [19](#__RefHeading___Toc354490696)

9. SAFETY REPORTING [19](#__RefHeading___Toc354490697)

9.1 Section 10 WMO event [19](#__RefHeading___Toc354490698)

9.2 AEs, SAEs and SUSARs [19](#__RefHeading___Toc354490699)

9.2.1 Adverse events (AEs) [19](#__RefHeading___Toc354490700)

9.2.2 Serious adverse events (SAEs) [19](#__RefHeading___Toc354490701)

9.3 Annual safety report [19](#__RefHeading___Toc354490702)

9.4 Follow-up of adverse events [20](#__RefHeading___Toc354490703)

9.5 Data Safety Monitoring Board (DSMB)/Safety Committee [20](#__RefHeading___Toc354490704)

10. STATISTICAL ANALYSIS [20](#__RefHeading___Toc354490705)

10.1 Primary study parameter(s) [20](#__RefHeading___Toc354490706)

10.2 Secondary study parameter(s) [20](#__RefHeading___Toc354490707)

10.3 Other study parameters [20](#__RefHeading___Toc354490708)

10.4 Analysis (if applicable) [20](#__RefHeading___Toc354490709)

11. ETHICAL CONSIDERATIONS [20](#__RefHeading___Toc354490710)

11.1 Regulation statement [20](#__RefHeading___Toc354490711)

11.2 Recruitment and consent [20](#__RefHeading___Toc354490712)

11.3 Objection by minors or incapacitated subjects (if applicable) [21](#__RefHeading___Toc354490713)

11.4 Benefits and risks assessment, group relatedness [21](#__RefHeading___Toc354490714)

11.5 Compensation for injury [21](#__RefHeading___Toc354490715)

11.6 Incentives (if applicable) [21](#__RefHeading___Toc354490716)

12. ADMINISTRATIVE ASPECTS, MONITORING AND PUBLICATION [21](#__RefHeading___Toc354490717)

12.1 Handling and storage of data and documents [21](#__RefHeading___Toc354490718)

12.2 Monitoring and Quality Assurance [21](#__RefHeading___Toc354490719)

12.3 Amendments [21](#__RefHeading___Toc354490720)

12.4 Annual progress report [21](#__RefHeading___Toc354490721)

12.5 End of study report [22](#__RefHeading___Toc354490722)

12.6 Public disclosure and publication policy [22](#__RefHeading___Toc354490723)

13. STRUCTURED RISK ANALYSIS [22](#__RefHeading___Toc354490724)

13.1 Potential issues of concern [22](#__RefHeading___Toc354490725)

13.2 Synthesis [22](#__RefHeading___Toc354490726)

**LIST OF ABBREVIATIONS AND RELEVANT DEFINITIONS**

| **ABR** | **ABR form (General Assessment and Registration form) is the application form that is required for submission to the accredited Ethics Committee (ABR = Algemene Beoordeling en Registratie)** |
| --- | --- |
| **AE** | **Adverse Event** |
| **AR** | **Adverse Reaction** |
|  |  |
| **CCMO** | **Central Committee on Research Involving Human Subjects** |
| **CV** | **Curriculum Vitae** |
|  |  |
| **EU** | **European Union** |
| **EudraCT** | **European drug regulatory affairs Clinical Trials GCP Good Clinical Practice** |
| **IB** | **Investigator’s Brochure** |
| **IC** | **Informed Consent** |
| **IMP** | **Investigational Medicinal Product** |
| **IMPD** | **Investigational Medicinal Product Dossier** |
| **METC** | **Medical research ethics committee (MREC); in Dutch: medisch ethische toetsing commissie (METC)** |
| **(S)AE** | **Serious Adverse Event** |
|  |  |
| **Sponsor** | **The sponsor is the party that commissions the organisation or performance of the research, for example a pharmaceutical company, academic hospital, scientific organisation or investigator. A party that provides funding for a study but does not commission it is not regarded as the sponsor, but referred to as a subsidising party.** |
| **SUSAR** | **Suspected Unexpected Serious Adverse Reaction** |
| **Wbp** | **Personal Data Protection Act (in Dutch: Wet Bescherming Persoonsgevens)** |
| **WMO** | **Medical Research Involving Human Subjects Act (Wet Medisch-wetenschappelijk Onderzoek met Mensen** |

**SUMMARY**

**Rationale:** To improve the rate of use of prosthetic devices in adults with an upper limb amputation, intermanual transfer might be helpful. Intermanual transfer is the ability to transfer motor skills from one, trained side to the other side (Hicks, 1983). This can be used in upper limb amputees by training the unaffected arm while waiting for the prosthesis to be fitted. Especially because it is assumed that starting to train early after the amputation will lead to better acceptance and improved prosthetic handling (Malone et al., 1984). By using intermanual transfer, the prosthetic skills of the affected arm will improve. Intermanual transfer effects were demonstrated to be present in myoelectric (Romkema, Bongers, & Van der Sluis, 2013) and body-powered prosthesis use (Weeks, Wallace, & Anderson, 2003). To further optimize the effects we compared the effect of training of different test tasks and the spacing over time in recent studies. The (preliminary) findings of these studies show that the intermanual transfer effects are small. We would like to add a mental imagery program to increase the effects of the intermanual transfer.

**Objective**: To establish whether mental imagery added to intermanual transfer has an larger effect on training than intermanual transfer alone. This will be done with 1) able-bodied participants using a prosthesis simulator, and 2) patients who start to use a prosthesis.

**Study design:** 1) experiment 1 is a single-blinded randomized trial, 2) experiment 2 is a case series.

**Study population:** 1) 48 non-amputated adults, 2) two amputees who will start to use the myo-electric prosthesis for the first time.

**Intervention (if applicable)**: 1) Two of three groups of 16 participants train to use a prosthesis simulator, one only using intermanual transfer and one using both intermanual transfer and mental imagery. A third group receives a sham training, without using the prosthesis simulator. In experiment 2, two patients with an amputation train (6 times 30 min) with the prosthesis simulator on the unaffected arm. The prosthesis simulator mimics the functioning of a real prosthesis but can be worn by able-bodied participants and at the sound arm of a patient with a unilateral upper limb amputation. The prosthesis simulator places a prosthesis hand in front of the sound hand.

**Main study parameters/endpoints:**

- Grip force control: mean deviation of the asked force in N.
- Movement time: time taken to execute the task in s.

**Nature and extent of the burden and risks associated with participation, benefit and group relatedness:**

The participants will use the prosthesis simulator to execute activities. This simulator mimics a real prosthesis device and can be worn over a sound arm. With the use of this simulator we are able to test more participants than only a few patients with an amputation who will start using a myoelectric prosthesis for the first time. Importantly, all the measurements are non-invasive and the use of a prosthesis simulator is not different from wearing a regular prosthesis. Therefore, the risks associated with participation can be considered negligible and the burden can be considered minimal.

# INTRODUCTION AND RATIONALE

To improve the rate of use of prosthesis devices in adults with an upper limb amputation we have been using intermanual transfer. Intermanual transfer implies that when you learn a motor task with one arm, not only that arm improves, but also the arm at the other side becomes better in the specific task (Hicks, Gualtieri, & Schoeder, 1983; Karni et al., 1998; Kumar & Mandal, 2005; Lee, Hinder, Gandevia, & Carroll, 2010; Mier & Petersen, 2006; Pereira, Raja, & Gangavalli, 2011). The untrained side thus benefits from the trained side. In other words, the effect of intermanual transfer is that the prosthesis skills of the affected arm will improve after training the unaffected side.

The intermanual transfer effect is shown to be present in patients using body-powered (Weeks et al., 2003) or myo-electric prostheses (Romkema et al., 2013). With able-bodied participants we showed that after training the ‘unaffected’ side using a prosthesis simulator, the level of skills at the ‘affected’ side increased. This effect can be useful in rehabilitation after an upper limb amputation, because the training can be started earlier. It is assumed that training immediately after the amputation will lead to better acceptance and prosthetic handling (Malone et al., 1984). It is found that training should start within one month after the amputation to achieve maximum success (Atkins, 1992; Dakpa & Heger, 1997; Gaine, Smart, & Bransby-Zachary, 1997). Though in this period often the wounds are not healed yet and the prosthesis should still be fabricated. Using a prosthesis simulator the training at the sound side can start early which may lead to better acceptance and higher prosthesis skills due to effects of intermanual transfer. This may decrease rejection rates of prosthesis devices.

The current study focuses on myo-electric prosthesis. To be able to train the unaffected arm we make use of a prosthesis simulator. With this simulator it is possible to mimic a myo-electric prosthesis. A prosthesis hand can be opened and closed with a motor driven by electrical signals that are produced by muscle activation. The simulator is placed over the arm, and the prosthesis hand is placed before the sound hand (Figure 1) and then operates in the same way as a prosthesis. The training with the prosthesis simulator is therefore comparable to the training with the myo-electric prosthesis.


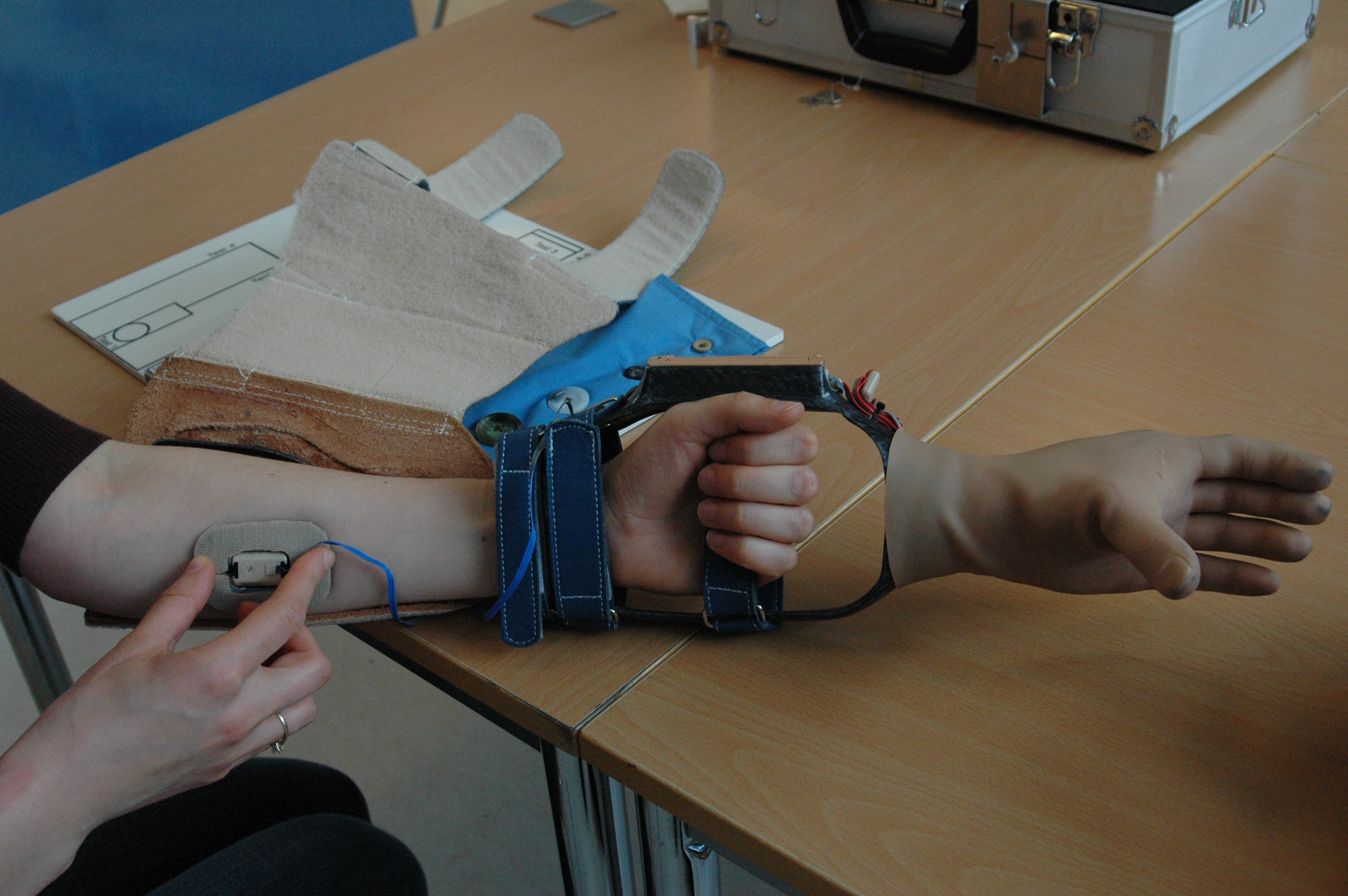


Figure 1. The prosthesis simulator.

In our earlier study (Romkema et al., 2013) where we demonstrated the effect of intermanual training in prosthesis learning we used functional tasks. We found that the movement times increased after a five-day training program. In following studies (data are analysed, papers are in preparation) we compared different training tasks and different time intervals between training sessions. Here we only found a minimal transfer effect for the training group on the functional and force control tasks.

Our results suggested that an important difference between the studies with effect and studies with hardly any effect, originate in the training the control group received. In studies in which we found an effect of intermanual transfer the control group received no training at all. In two other studies we found a minimal or no effect at all of the intermanual transfer training. In that study we used a sham group training an unrelated motor skill. Training this motor skill might have resulted in neural activity that improved skills with the untrained hand, thereby masking differences between the training and control group.

From these findings one might conclude that even though there is intermanual transfer, the effects are limited. Therefore in the coming study we will try to enlarge the transfer effect by adding mental imagery using functional and force control tasks.

To be able to examine the effects of the additional therapy, participants will be tested in a pre-test, post-test, retention test design. This means that independent of the training received, all participants will perform the tests. The goal of this study is to establish the additional benefit of intermanual transfer when combined with mental imagery above the effect of intermanual transfer alone.

*Mental Imagery*

We will use two kinds of mental imagery, namely mirror therapy and motor imagery. Mirror therapy involves movement of one hand in front of a mirror, such that visual feedback of the other hand is replaced by the reflection. (Moseley, 2004) Motor imagery is the imagining of a movement of the hand without actually moving it. (Boonstra et al., 2012; Decety, 1996)

Mental imagery originally was used to treat pain or relearn sensibility (Ramachandran & Rogers-Ramachandran, 1996). Though, our aim is not to treat pain, but to improve motor learning. Literature focusing on the effect of mental imagery on motor learning is showing some positive effects. Mirror therapy has shown effects in stroke patients (Thieme, Mehrholz, Pohl, Behrens, & Dohle, 2012). By using the mirror an increase of the activity of the hemisphere of the affected arm is shown (Garry, Loftus, & Summers, 2005). Motor imagery has shown some positive effects in stroke patients (Altschuler et al., 1999; Page, Levine, & Leonard, 2007) and other diseases (Schuster et al., 2011).

Also in training of athletes it is found that learning can be accelerated and performance can be improved when using mental practice (Feltz & Landers, 1983; Lotze & Halsband, 2006; Saimpont et al., 2013).

We will combine intermanual transfer with mental imagery to improve motor skills. To our knowledge this has never been done before. Other studies including intermanual transfer and (parts of) mental imagery mostly focus on the transfer of force from one limb to the other. In a theoretical (Howatson, Zult, Farthing, Zijdewind, & Hortobagyi, 2013) and review (Zult, Howatson, Kadar, Farthing, & Hortobagyi, 2013) article it is predicted that mirror therapy is more effective than intermanual transfer alone for resistance training. In another study on resistance training Farthing et al. (2007) used, apart from the training group that trained force, a control group that was trained using motor imagery. They tested the effects of motor imagery on strength, though did not find an effect. Though, the imagery group did show changes in brain activation in regions that were somewhat similar to the training group.

Other articles including both strategies focus on the occurrence of intermanual transfer after motor imagery training. Thus, one side is trained using motor imagery, while the other side is tested. For a tapping task it was found that “execution was effective especially for trained movement, imagery was effective for both trained movement and intermanual transfer.“ (Amemiya, Ishizu, Ayabe, & Kojima, 2010) The same conclusion was drawn from a study using Box and Block test (Greiner, Schoenfeld, & Liepert, 2014).

Thus mental imagery, mirror therapy as well as motor imagery, is beneficial in motor learning. The combination of intermanual transfer and mental imagery is only studied in experiments focusing on force, though is found to be beneficial. We therefore assume mental imagery could be beneficial in prosthetic training. Below we describe how we would like to add mental imagery to our training.

Mirror therapy

Mirror therapy is added to extend intermanual transfer training with visual feedback. While the unaffected hand is trained using a prosthesis simulator the arm is projected in a mirror so that the participant sees a reflection of the unaffected arm (figure 2). In this manner it seems that the (‘affected’) test arm is executing the same movements. The mirror gives the participant the illusion that the arm is present and this is used to give feedback about the movements of the prosthesis hand.


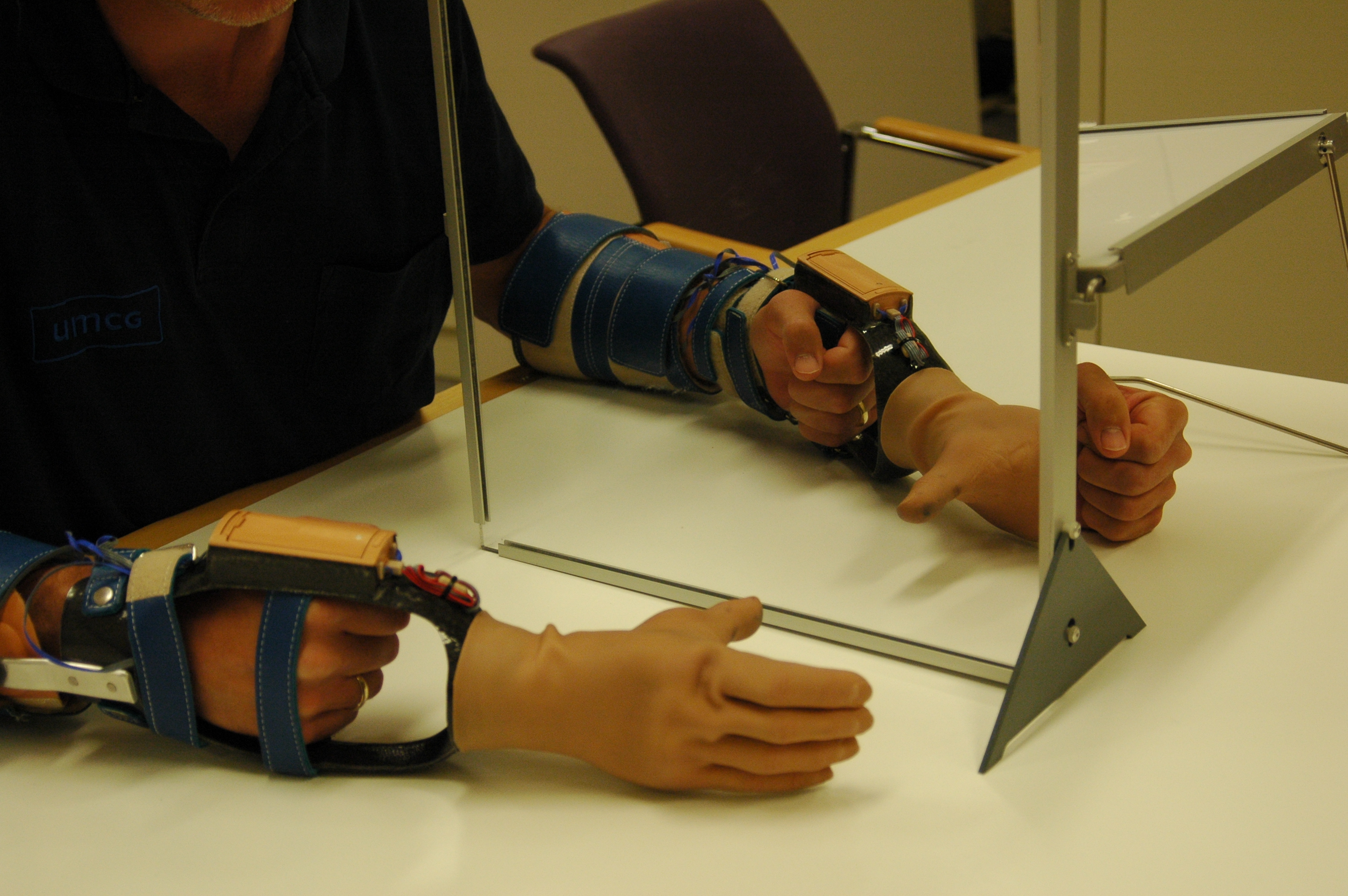


Figure 2. Intermanual transfer extended with mirror therapy.

Motor imagery

For controlling the prosthesis simulator, the wrist muscles are used. Therefore participants need to be able to imagine making flexion and extension movements of the wrist. Literature states that in order to activate the right brain parts ‘a well-established motor representation from physical training is required’. (Olsson & Nyberg, 2010) It is unclear if it is enough to be able to move the wrist or that the participant needs to be able to control a prosthesis. As such, we would like the participants to become familiar with handling a prosthesis before starting motor imagery. Therefore the training needs to be started using the prosthesis simulator on the unaffected side. Once the simulator can be controlled the participant can start training using motor imagery. In this training the participant has to imagine to use the prosthesis at the unaffected and affected side.

For the characteristics of the training the article of Schuster et al. will be used. They describe the most effective aspects of motor imagery according to the PETTLEP aspects (Holmes, 2001) aspects. PETTLEP is an imagery framework from sport literature based on findings of functional neuroscientific literature and experience in sport psychology. The model consists of seven components (physical, environment, task, timing, learning, emotion and perspective). These components describe for example the physical position of the individual, the task involved, or the timing of the imagery. As such, for the largest effect it should be expected that the participants not only imagine the movement though imagine that activities are executed using a prosthesis.

In conclusion, with this study we aim to enlarge the intermanual transfer effects in prosthetic use by adding mental imagery to a training program using intermanual transfer.

The first experiment will be performed with able-bodied participants. Including able-bodied participants means that we do not have to bother patients who have just been amputated. If it will be possible to improve intermanual transfer effects this might have consequences for the training program of patients with traumatic amputations. In the second experiment we will try to generalize the findings to patients.

# OBJECTIVES

To establish whether mental imagery added to intermanual transfer has an larger effect on training than intermanual transfer alone. This will be done with 1) able-bodied participants using a prosthesis simulator, and 2) patients who start to use a prosthesis.

# STUDY DESIGN

The designs of the experiments are presented in Tables 1 and 2. Based on one of our earlier studies we decided to train the participants on five consecutive days (Romkema et al., 2013).

Experiment 1 (Table 1)

The goal of the first experiment is to test if intermanual transfer effects of able-bodied adults using the simulator can be increased. There will be two training groups. The participants in these groups learn to use the simulator on one arm (training arm). The other arm (test arm) is tested to assess if there is an intermanual transfer effect. One group will only obtain intermanual transfer training while the other group obtains intermanual transfer training extended with mental imagery. Both training groups will get a functional training and a training focused on force control. The control group receives a sham training not consisting of motor learning. All training programs take 45 minutes per session and are executed on five consecutive days.

The tests comprise a pretest, posttest and retention test (six days after ending the training), to be able to measure whether there are learning effects and whether these effects remain (see 7.3 for an extensive explanation). All tests consist of the same tasks; functional and grip force control tasks. Half of the participants will train their dominant hand and half will train their non-dominant hand.

Table 1. Study design of the first experiment.

| **Healthy adults** | | | | | | |
| --- | --- | --- | --- | --- | --- | --- |
| *Training* | **Intermanual transfer** | | **Intermanual transfer and mental imagery** | | **Sham training** | |
| *Participants* | *4 men dom; 4 men n-dom, 4 women dom; 4 women n-dom* | | *4 men dom; 4 men n-dom, 4 women dom; 4 women n-dom* | | *4 men dom; 4 men n-dom, 4 women dom; 4 women n-dom* | |
| **Day** | **Test** | **Practice** | **Test** | **Practice** | **Test** | **Practice** |
| 1 | Pretest | 45 min | Pretest | 45 min | Pretest | 45 min |
| 2 | - | 45 min | - | 45 min | - | 45 min |
| 3 | - | 45 min | - | 45 min | - | 45 min |
| 4 | - | 45 min | - | 45 min | - | 45 min |
| 5 | Posttest | 45 min | Posttest | 45 min | Posttest | 45 min |
| 11 | Retention test | - | Retention test | - | Retention test | - |

Experiment 2 (Table 2)

The design of the second study is shown in table 2. This experiment aims to reveal the effect of intermanual transfer with mental imagery in patients. For this experiment patients from the UMCG with an amputation, who will get a myo-electric prosthesis for the first time, will be included. The design of the experiment is comparable to that of the one described above. The two patients will get intermanual transfer training extended with mental imagery. The results of these patients will be compared to a) the results of patients who did not receive any additional intermanual training, though they did perform the tests and b) the results from patients who received intermanual transfer only. These patients were included in our earlier studies (NL35268.042.11, NL43335.042.13). The pretest is left out for all patients, because it is impossible due to the amputation.

Table 2. Study design of the second experiment in patients.

| Patients |  | |
| --- | --- | --- |
| *Training* | Intermanual transfer with mental imagery | |
| *Participants* | 2 amputees | |
| **Session** | **Test** | **Practice** |
| 1 | - | 45 min |
| 2 | - | 45 min |
| 3 | - | 45 min |
| 4 | - | 45 min |
| 5 | Posttest | 45 min |
| 13 | Retention test | - |
| 20 | Retention test | - |

# STUDY POPULATION

## Population (base)

(1) 48 non-amputated adults and (2) 2 patients with an acquired upper limb amputation who will obtain a myo-electric prosthesis for the first time.

## Inclusion criteria

*Able-bodied persons*

(1) Normal or corrected to normal sight

(2) Right-handed

(3) Aged 18 till 40

*Patients*

(1) An unilateral forearm amputation and an indication for a first myo-electric prosthesis

## Exclusion criteria

*Able-bodied persons*

1. Neurological problems concerning upper extremity or torso
2. Motor problems concerning upper extremity or torso
3. Earlier experience with a prosthesis simulator
4. Limited sight despite correction

*Patients*

1. An upper limb amputation at a different level than a forearm amputation
2. Insufficient knowledge of the Dutch language
3. Inability to follow instructions
4. Diseases affecting the joints, nerves or muscles of the non- amputated arm, such as rheumatoid arthritis.

All criteria are based on what the participants tell us

## Sample size calculation

To establish the number of adult able-bodied participants that should be included for the first experiment we used the data of an earlier study (NL43335.042.13, experiment 1). In both experiments, the tests and training schedule (5 consecutive days) are the same. We used the pretest and retention test of the force control task to decide what the expected differences are. Using g power we made an estimation of the amount of participants necessary within each group to reach a power of 0.8. Here for we used a t-test based on differences between two dependent means. The type of the power analysis is ‘a priori’, we used double side testing with an alfa of 0.05.

Apart from the similarities there are also some differences between the experiments. Because of these changes we cannot give an exact estimation. The training will be longer and the tests shorter. We therefore expect the effects to be somewhat larger.

With the described method we found that we need to have 14 participants in each group (Table 1). We would like to include 16 participants to get an equal distribution of men/women and dominant/not dominant test hand per group.

| **Sample size per group** | |  | | |  | |  | |
| --- | --- | --- | --- | --- | --- | --- | --- | --- |
|  |  | | power | pretest | | retentie test | |  |
| taak | effect size | | 0.8 | Deviatie (SD) | | Deviatie (SD) | |  |
| force control | .89 | | 17 | 7.39 (3.28) | | 5.04 (1.76) | |  |

Tabel 1

For the second experiment with the patients we are depending on the amount of participants that are available and willing to participate. We need to find participants who recently had an arm amputation and are waiting for a myo-electric prosthesis. We aim to include two prosthetic users. This is the number of participants we expect to be able to include during the running of the experiment. Of course this amount of patients is not enough to perform statistical tests. This data is therefore only used as case studies of which descriptive statistics will be presented. With that, including two patients enables us to compare the data with data of an earlier studies on patients. (NL35268.042.11 and NL43335.042.13)

# TREATMENT OF SUBJECTS

## Investigational product/treatment

The participants learn to use the prosthesis simulator during training sessions. These sessions take place on five days to promote learning. Each training session will take thirty minutes.

## Use of co-intervention (if applicable)

Not applicable

## Escape medication (if applicable)

Not applicable

# INVESTIGATIONAL PRODUCT

Not applicable

# NON-INVESTIGATIONAL PRODUCT

Not applicable

# METHODS

## Study parameters/endpoints

### Main study parameter/endpoint

- Grip force control: mean deviation of the asked force in N is measured in the grip force control tasks.
- Movement time: time taken to execute the movement in seconds is measured in the functional task.

### Secondary study parameters/endpoints (if applicable)

Not applicable

### Other study parameters (if applicable)

Not applicable

## Randomisation, blinding and treatment allocation

The able-bodied adults will be randomly assigned to one of the three groups. The number of participants that train with their dominant and non-dominant hand will be equal for both sexes. The researcher will be blinded for the experiments, the participants cannot be blinded.

## Study procedures

Prior to the start of the experiment all participants sign an informed consent and it will be explained to them that they can stop with the experiment at any time, without giving a reason.

Design tests and training

To measure the effect of additional MENTAL IMAGERY we compare the results of this training group with a group who only obtains intermanual transfer training. The training programs are both focusing on learning to execute functional tasks and to control force. After executing one of the training programs the intermanual transfer effect on the functional and force control skills is measured. With the results it is possible to measure the difference between only intermanual transfer or intermanual transfer extended with mental imagery.

In the experiment the tasks executed during the training sessions and tasks executed during the tests differ from each other. This is to resemble a rehabilitation setting. That is, what needs to be learned in not executing a single task with the prosthesis, but the skill of using the prostheses in a wide variety of tasks needs to be learned. In choosing the tasks for the training and test sessions we also take into account the task complexity. A relative complex task is used for the training because then the effects on the other arm (when executing a simpler task) are assumed to be more prominent.

Materials

The myo-electric simulator is developed to closely resemble a myo-electric upper extremity prosthesis for a below-elbow amputation (see Figure 1). The simulator consists of a myo-electric hand, the MyoHand VariPlus Speed® of Otto Bock, attached to an open cast in which the hand can be placed. The cast extends into a splint along the forearm, adjustable in length. The splint can be attached to the arm using a self-adhesive (Velcro) sleeve. The prosthesis hand has proportional speed control (15-399 mm/s) and proportional grip force control (0-±100 N). The hand is controlled by changes in electric muscle activity, detected by 2 electrodes that are placed on the extensors and flexors in the forearm. The exact positions of these electrodes are determined after palpation of the most prominent contraction of muscle bellies of the extensors and flexors. Subsequently, these locations are marked to place the electrodes. The position of the electrodes is then optimized using Otto Bock PAULA®. Hand opening is accomplished by activity of the extensors, while the hand is closed by activity of the flexors.


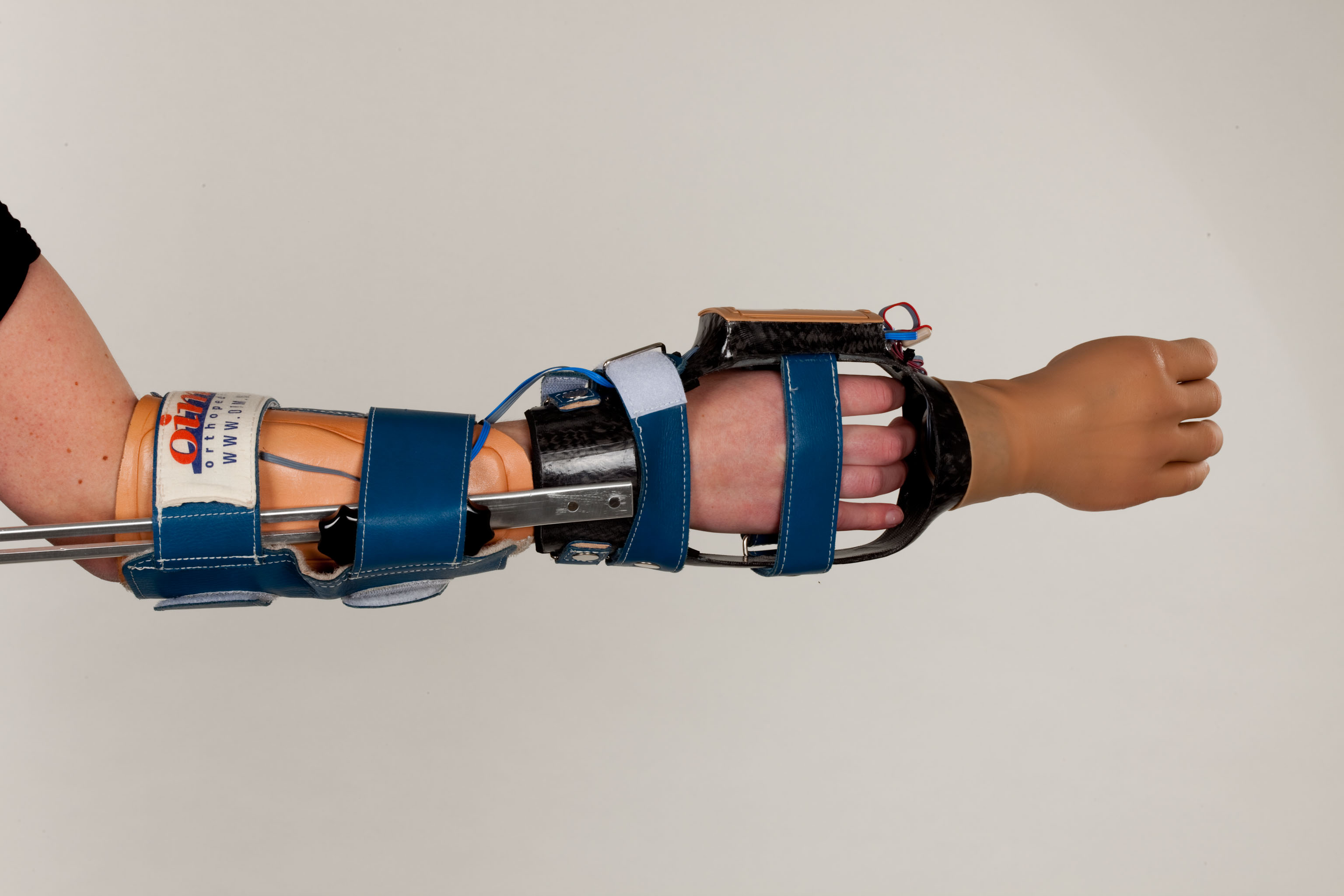

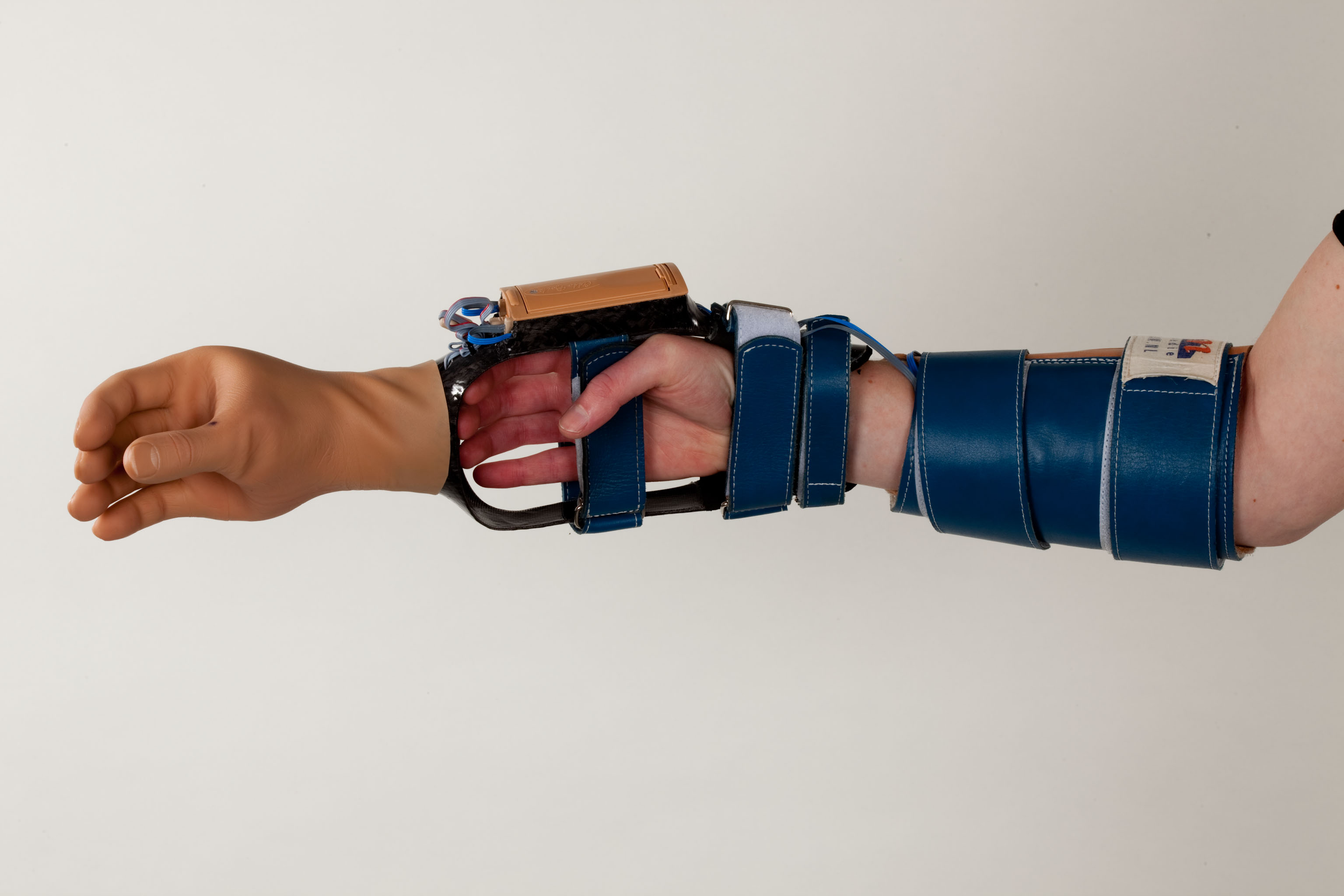

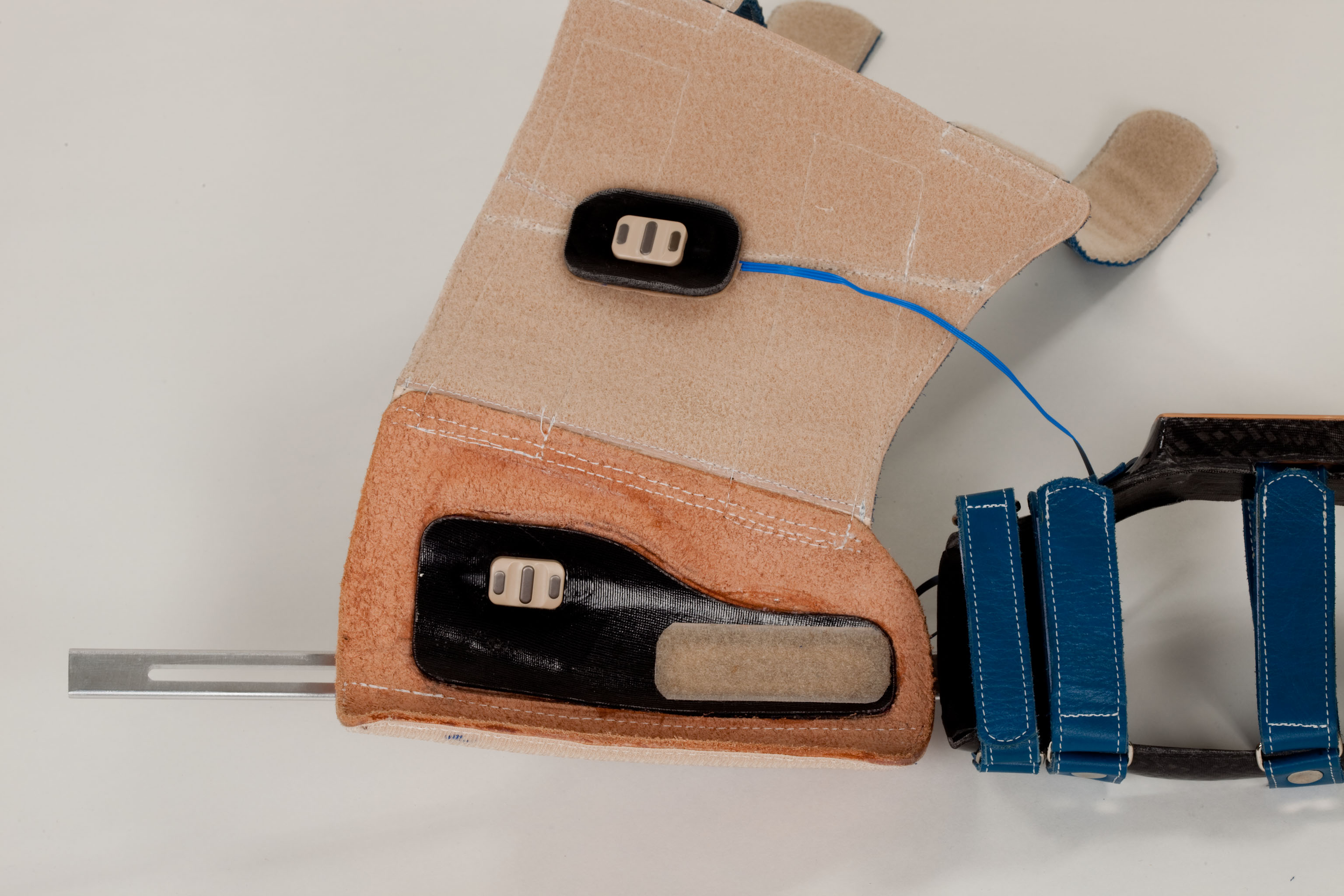
’

*Figure 1 a, b and c.* The myo-electric simulator, dorsal, volar side and the electrodes inside.

The 757M11 MyoBoy® is used for the fitting of the electrodes of the simulator. It is used to evaluate the sensitivity of the myo-signals.

The Southampton Hand Assessment Procedure (SHAP) is a standardized test to measure prosthetic handling, consisting of 12 abstract and 14 functional tasks. Tasks of the SHAP are used for functional training.

PFOR is a custom made program to measure the amount of force when pinching a handle. The handle consist of two plates with a load sensor placed in between that measures the amount of force (Figure 2). In the task, the participant has to track a line that will be shown on the screen. The asked and performed forces are shown on the screen. We have had approval of the department of ‘Medische Techniek’ to use this program.

Figure 2. PFOR with handle.

A mirror that can stand in a vertical position is used for the mirror therapy.

Procedure

Before the first measurement the Edinburgh Handedness Inventory (Oldfield, 1971) is filled in; only right handed participants are included. This questionnaire consists of 10 Items and it takes 1 minute to fill in.

Before each training and test session, a standard protocol is conducted in order to fit the simulator. The simulator is fitted with help of Otto Bock MyoBoy®. The electrodes within the simulator have to be placed on the optimal locations. The settings need to be tailored to each individual in order to record the myoelectric signal properly. The sensitivity of the electrodes will be adjusted for each participant on each day, so that all participants can just reach the myoelectric threshold of 1.5 V (high signal) and hold it for 2 seconds. The maximum speed of the hand is set to the default setting of 6 (range = 1-6). After the simulator is installed, the participant will be seated at a table and the session will start.

Prior to each task, during the training as well as the tests, the experimenter gives the participant instructions to execute the task. The participants are told to sit comfortably at a table, with their arms resting on the table. They start each task with the prosthesis hand closed. The participants are instructed to execute the tasks as rapidly as possible.

*Tests*

The test tasks differ from the training tasks, though they are based on the same skills (functional and force control). The two test tasks (one for each skill) are used to administer the pretests, posttests and one retention tests. Below, the two test tasks are described in detail. During the tests the simulator is worn on the test hand (the ‘affected’ hand). During training the simulator is worn on the other, ‘unaffected’ hand.

Functional test tasks

The functional test tasks consist of three object manipulation tasks, as described by Bouwsema (2008). The tasks are based on the three different ways the prosthesis is handled according to van Lunteren et al. (van Lunteren, van Lunteren-Gerritsen, Stassen, & Zuithoff, 1983); direct grasping, indirect grasping and fixating. In the ‘pick-up mug task’ the participant has to pick up a mug at the handle with the simulator and to place it 25 cm above the table on a shelf. In the ‘lid-off jar task’ a jar is picked up by the sound hand at the start and has to be handed over to the simulator, the lid had to be removed by turning it with the sound hand. In the ‘zipper task’ a pencil case is hold with the simulator at the start position and then the zipper is opened with the sound hand.

The dependent variable measured in these tests is the movement time, the time taken to execute the movement in seconds and the hand opening.

Grip force control test tasks

Grip force control in this study is the control of the grip force executed on an object that is grasped by the prosthesis hand. Intermanual transfer of force control is found to be possible, though it seems to depend on the situation. Maximum force is shown to transfer in different studies, for example the handgrip strength of an immobilized hand can be remained (Farthing et al., 2011) and the maximal acceleration of finger abduction (a form of maximal force) is improved after training. (Lee et al., 2010) Transfer of proportional isometric force has only been shown between limbs on two sides of the body, not between upper and lower limbs (Christou & Rodriguez, 2008). Control of force referred towards the environment (Bensmail, Sarfeld, Fink, & Nowak, 2010; Chang, Flanagan, & Goodale, 2008; Teixeira, 2000), like in lifting objects, showed better intermanual transfer results than force referred towards maximal output, like giving a percentage of the maximum force (Christou & Rodriguez, 2008; Park & Shea, 2002).

We will therefore use a tracking task (see 8.3 Study procedures, Materials). In the tracking task a pattern on the screen needs to be followed for 30 seconds by pressing a handle with the prosthesis hand (Figure 4). The pattern consists of different levels of absolute forces (ranged 5 – 45 N) that vary in a blocked pattern. Each amount of force needs to be hold for two seconds. After each trial the participant is allowed to take a break for a few seconds. The course of the pattern appears slightly (200 ms) before the subject has to produce the force. The pattern starts with a line of three seconds of a force of ten Newton, to make sure that participants are able to position the prosthesis hand on the handle, and that all participants have the same starting position. After these first three seconds the block pattern starts.

The dependent variable for this test is the mean deviation of the asked force in N.

*Figure 4*. The line of the asked force (yellow) and produced force (red) that can be seen on the computer screen during the tracking task.

*Pretest*

At the first day, the two test tasks are administered to determine the level of skills of the participants of the experimental and control groups. The test is performed with the test hand.

*Posttest and retention test*

All participants perform a posttest, equal to the pretest. Six days after the posttest and these participants execute a retention test. For both tests the participants again perform the two test tasks, in order to determine the improvement of skills and compare the different groups. The tasks are once more presented in a randomized order and executed with the test hand.

*Training*

During the training sessions the simulator is worn on the training hand (‘unaffected’ hand). All training programs are executed during 45 minutes. There are two experimental groups and one control group.

Mental imagery training group

1. *Intermanual transfer (30 minutes)*

For thirty minutes a day the participants trains to use the prosthesis simulator using intermanual transfer. The training consist of functional tasks and force control tasks. The functional tasks are trained during twenty minutes using all tasks of the SHAP. The force control tasks are trained during 10 minutes using the tracking task (see the above).

Furthermore, the mental imagery is trained during fifteen minutes:

1. *Mirror therapy (10 minutes)*

After training the movements with the ‘unaffected’ hand the mirror therapy is started. The mirror therapy will consist of reflection of the hand with the prosthesis simulator with the arm in rest, with the arm moving and while moving the prosthesis hand. After this, the participants are asked to execute simple unilateral tasks (like picking up a mug). At the start the participant is asked to just look at the reflection, later on the participant is asked to imagine that the reflection is the ‘affected’ hand.

During all the mirror therapy the participant is asked to keep the ‘affected’ hand inactive. This is controlled visually by the researchers.

1. *Motor imagery (5 minutes)*

During the motor imagery the participants are asked to imagine movements of the ‘affected’ hand using a simulator. During the imagining the participants are asked not to move, this is again visually controlled. The participant is asked to imagine to wear the prosthesis themselves, then they are asked to open en close the hand, to control the opening and closing and at last to execute simple unilateral activities.

Intermanual transfer training group

This group gets the same intermanual transfer training as the mental imagery group, again during 30 minutes. Besides this they will get a sham training during 15 minutes to keep the training time equal. Our earlier studies indicate an effect of sham training when a motor skill was trained. Therefore we now will let them play the game Patience with playing cards.

Control group

The control group is executing a sham training for a period of time that the other groups train (45 minutes). They will make Sudoku puzzles for 30 min and play Patience for 15 min. To measure performance we count the amount of Sudoku’s made and we will count the cards left in the remaining pack.

## Withdrawal of individual subjects

Participants can leave the study at any time for any reason if they wish to do so without any consequences. The investigator can decide to withdraw a participant from the study for urgent medical reasons. Since the experiments are safe, we do not expect any urgent medical occasions.

### Specific criteria for withdrawal (if applicable)

Not applicable

## Replacement of individual subjects after withdrawal

If a participant withdraws from the study, another participant will be asked to join the study, preferably of the same sex.

## Follow-up of subjects withdrawn from treatment

Not applicable

## Premature termination of the study

Not applicable

# SAFETY REPORTING

## Section 10 WMO event

In accordance to section 10, subsection 1, of the WMO, the investigator will inform the participants and the reviewing accredited METC if anything occurs, on the basis of which it appears that the disadvantages of participation may be significantly greater than was foreseen in the research proposal. The study will be suspended pending further review by the accredited METC, except insofar as suspension would jeopardise the participants’ health. The investigator will take care that all participants are kept informed.

## AEs, SAEs and SUSARs

### Adverse events (AEs)

Adverse events are defined as any undesirable experience occurring to a subject during the study, whether or not considered related to the experimental intervention (e.g. prosthesis simulator training or testing). All adverse events reported spontaneously by the subject or observed by the investiga­tor or his staff will be recorded.

### Serious adverse events (SAEs)

A serious adverse event is any untoward medical occurrence or effect that at any dose:

results in death;

- results in death;
- is life threatening (at the time of the event);
- requires hospitalisation or prolongation of existing inpatients’ hospitalisation;
- results in persistent or significant disability or incapacity;
- is a congenital anomaly or birth defect;
- Any other important medical event that may not result in death, be life threatening, or require hospitalization, may be considered a serious adverse experience when, based upon appropriate medical judgement, the event may jeopardize the subject or may require an intervention to prevent one of the outcomes listed above.

Any other important medical event that may not result in death, be life threatening, or require hospitalization, may be considered a serious adverse experience when, based upon appropriate medical judgement, the event may jeopardize the subject or may require an intervention to prevent one of the outcomes listed above.

The investigator is responsible for reporting all SAE’s to the sponsor. This is independent of the centre where the research takes place. The sponsor will report the SAEs through the web portal ToetsingOnline to the accredited METC that approved the protocol, within 15 days after the sponsor has first knowledge of the serious adverse reactions.

SAEs that result in death or are life threatening should be reported expedited. The expedited reporting will occur not later than 7 days after the responsible investigator has first knowledge of the adverse reaction. This is for a preliminary report with another 8 days for completion of the report.

## Annual safety report

Not applicable

## Follow-up of adverse events

Not applicable

## Data Safety Monitoring Board (DSMB)/Safety Committee

Not applicable

# STATISTICAL ANALYSIS

## Primary study parameter(s)

Deviation of grip force control,

Plateau phase in hand opening

Movement time and initiation time.

## Secondary study parameter(s)

Age

Gender

Additional parameters for the patients:

Amputation side,

Dominant side,

Months between the amputation and obtaining the prosthesis

Hours of therapy

## Other study parameters

Not applicable

## Analysis (if applicable)

In both experiments all measurements (deviation of grip force control, hand opening (length of plateau phase in grasping), movement time and initiation time) are subjected to a repeated-measures ANOVA with test (pre-test, post-test and retention test) as within-subject factor and dominance (preferred, non-preferred) and group (two experimental groups and one control group;) as between-subject factors. In the second experiment, the same effects are analyzed.

In the second experiment we analyse the univariate statistics of the additional parameters.

When a Mauchly test indicates that sphericity is violated, the degrees of freedom are adjusted with the Greenhouse-Geisser correction. In all analyses, a significant criterion of α less than or equal to 0.05 is used, and post hoc tests on main effects use Bonferroni adjustment.

# ETHICAL CONSIDERATIONS

## Regulation statement

The study will be conducted according to the principles of the Declaration of Helsinki (64th, October 2013) and in accordance with the Medical Research Involving Human Subjects Act.

## Recruitment and consent

The able-bodied participants will be recruited by advertisement on publication boards of different faculties of the University of Groningen, and information presented in course lectures of Human Movement Sciences and Medicine by the investigator. To include the patients, physicians from the Department of Rehabilitation Medicine UMCG are informed about this study and asked to inform patients. Patients will be told that this study is conducted and asked whether a researcher may contact them for information about the study.

The participants will receive an information letter, with written information about the experiment, after they have shown interest in participating in the experiment. Participants will get between 1 and 8 weeks to decide whether they would like to join the study. For each potential participant there is the possibility to consult the researcher or an independent physician for any further information, this is also mentioned in the letter. After participants have signed in, they will sign an informed consent before the start of the experiment and it will be explained to them that they can stop with the experiment at any time without giving a reason. This can be done by telling the researchers.

## Objection by minors or incapacitated subjects (if applicable)

Not applicable

## Benefits and risks assessment, group relatedness

The participants will learn to use a simulator during training sessions and will be tested on their abilities. All training sessions are done with non-injured hands and the measurements are non-invasive. Therefore, the risks associated with participation can be considered negligible and the burden can be considered minimal.

## Compensation for injury

Because participation in the experiment is without risks, the judging committee, the METc UMCG has granted a release from compulsory insurance, as referred to in section 4 paragraph 1 of the ‘Besluit verplichte verzekering bij medisch-wetenschappelijk onderzoek met mensen’.

## Incentives (if applicable)

Not applicable

# ADMINISTRATIVE ASPECTS, MONITORING AND PUBLICATION

## Handling and storage of data and documents

The data is handled confidentially and coded for each participant; each participant will be given a number from 1 to 48. The investigator will keep the data for the duration of the project. The handling of personal data is complied with the Dutch Personal Data Protection Act (De Wet Bescherming Persoonsgegevens, Wbp).

## Monitoring and Quality Assurance

Monitoring of the conduct of the study takes place by J.M. Hijmans. The aim is to verify the rights and well-being of the participants, to check if the reported information is correctly derived from the original data and if the execution of the experiment in consensus is with the protocol, with good clinical practice and relevant laws. The inclusion of participants, the possible advents, the execution of the study and the progress of the study are monitored. The monitoring takes place at least once a week and can take place in the lab where the experiments are conducted.

## Amendments

Amendments are changes made to the research after a favourable opinion by the accredited METC has been given. All amendments will be notified to the METC that gave a favourable opinion.

The amendments will be implemented after the METc gives a positive judgement.

Non-substantial amendments will not be notified to the accredited METC and the competent authority, but will be recorded and filed by the sponsor.

## Annual progress report

The investigator will submit a summary of the progress of the trial to the accredited METC after a year. Information will be provided on the date of inclusion of the first subject, numbers of subjects included and numbers of subjects that have completed the trial, serious adverse events/ serious adverse reactions, other problems and amendments.

## End of study report

The investigator will notify the accredited METC of the end of the study within a period of 8 weeks. The end of the study is defined as the last patient’s last visit.

In case the study is ended prematurely, the investigator will notify the accredited METC, including the reasons for the premature termination.

Within one year after the end of the study, the investigator/sponsor will submit a final study report with the results of the study, including any publications/abstracts of the study, to the accredited METC.

## Public disclosure and publication policy

The results of this study will be unreservedly published in a peer reviewed scientific journal.

# STRUCTURED RISK ANALYSIS

In this study a medical device, e.g. the prosthesis simulator, will be used.

## Potential issues of concern

In this study we will use a medical device, namely the prosthesis simulator. We expect that this study has no risk of potential issues of concern for the participants. The used prosthesis simulator is an approved medical device (approval is added). Furthermore, the device is used before in several experiments of our study group (NL26993.042.09, NL35268.042.11 and NL43335.042.13).

## Synthesis

Not applicable

REFERENCES

[1] J. M. Malone, L. L. Fleming, J. Roberson, T. E. Whitesides Jr, J. M. Leal, J. U. Poole and R. S. Grodin, "Immediate, early, and late postsurgical management of upper-limb amputation,"  *J.  Rehabil.  Res.  Dev.,* vol. 21, pp. 33-41, May, 1984.

[2] S. Romkema, R. M. Bongers and C. K. Van der Sluis, "Intermanual transfer in training with an upper-limb myoelectric prosthesis simulator: a mechanistic, randomized, pretest-posttest study,"  *Phys.  Ther.,* vol. 93, pp. 22-31, Jan, 2013.

[3] D. L. Weeks, S. A. Wallace and D. I. Anderson, "Training with an upper-limb prosthetic simulator to enhance transfer of skill across limbs,"  *Arch.  Phys.  Med.  Rehabil.,* vol. 84, pp. 437-443, Mar, 2003.

[4] R. E. Hicks, C. T. Gualtieri and S. R. Schoeder, "Cognitive and motor components of bilateral transfer,"  *American Journal of Psychology,* vol. 96, pp. 223-228, Jul, 1983.

[5] A. Karni, G. Meyer, C. Rey-Hipolito, P. Jezzard, M. M. Adams, R. Turner and L. G. Ungerleider, "The acquisition of skilled motor performance: fast and slow experience-driven changes in primary motor cortex,"  *Proc.  Natl.  Acad.  Sci.  U.  S.  A.,* vol. 95, pp. 861-868, Feb 3, 1998.

[6] S. Kumar and M. K. Mandal, "Bilateral transfer of skill in left- and right-handers,"  *Laterality,* vol. 10, pp. 337-344, 2005.

[7] M. Lee, M. R. Hinder, S. C. Gandevia and T. J. Carroll, "The ipsilateral motor cortex contributes to cross-limb transfer of performance gains after ballistic motor practice,"  *Journal of Physiology,* vol. 558, pp. 201-212, 2010.

[8] H. I. Mier and S. E. Petersen, "Intermanual transfer effects in sequential tactuomotor learning: Evidence for effector independent coding,"  *Neuropsychologia,* vol. 44, pp. 939-949, 2006.

[9] E. A. Pereira, K. Raja and R. Gangavalli, "Effect of training on interlimb transfer of dexterity skills in healthy adults,"  *Am.  J.  Phys.  Med.  Rehabil.,* vol. 90, pp. 25-34, Jan, 2011.

[10] D. Atkins, "Adult upper limb prosthetic training," in *Atlas of Limb Prosthetics: Surgical, Prosthetic, and Rehabilitation Principles*, 2nd ed., H. K. Bowker and J. W. Michael, Eds. Rosemont, I.L., 1992, .

[11] R. Dakpa and H. Heger, "Prosthetic management and training of adult upper limb amputees,"  *Current Orthopaedics,* vol. 11, pp. 193-202, 1997.

[12] W. J. Gaine, C. Smart and M. Bransby-Zachary, "Upper limb traumatic amputees. Review of prosthetic use,"  *J.  Hand Surg.  Br.,* vol. 22, pp. 73-76, Feb, 1997.

[13] G. L. Moseley, "Graded motor imagery is effective for long-standing complex regional pain syndrome: a randomised controlled trial,"  *Pain,* vol. 108, pp. 192-198, Mar, 2004.

[14] A. M. Boonstra, S. J. de Vries, E. Veenstra, M. Tepper, W. Feenstra and E. Otten, "Using the Hand Laterality Judgement Task to assess motor imagery: a study of practice effects in repeated measurements,"  *Int.  J.  Rehabil.  Res.,* vol. 35, pp. 278-280, Sep, 2012.

[15] V. S. Ramachandran and D. Rogers-Ramachandran, "Synaesthesia in phantom limbs induced with mirrors,"  *Proc.  Biol.  Sci.,* vol. 263, pp. 377-386, Apr 22, 1996.

[16] L. Cossins, R. W. Okell, H. Cameron, B. Simpson, H. M. Poole and A. Goebel, "Treatment of complex regional pain syndrome in adults: a systematic review of randomized controlled trials published from June 2000 to February 2012,"  *Eur.  J.  Pain,* vol. 17, pp. 158-173, Feb, 2013.

[17] K. J. Bowering, N. E. O'Connell, A. Tabor, M. J. Catley, H. B. Leake, G. L. Moseley and T. R. Stanton, "The effects of graded motor imagery and its components on chronic pain: a systematic review and meta-analysis,"  *J.  Pain,* vol. 14, pp. 3-13, Jan, 2013.

[18] E. E. Brodie, A. Whyte and C. A. Niven, "Analgesia through the looking-glass? A randomized controlled trial investigating the effect of viewing a 'virtual' limb upon phantom limb pain, sensation and movement,"  *Eur.  J.  Pain,* vol. 11, pp. 428-436, May, 2007.

[19] E. L. Altschuler, S. B. Wisdom, L. Stone, C. Foster, D. Galasko, D. M. Llewellyn and V. S. Ramachandran, "Rehabilitation of hemiparesis after stroke with a mirror,"  *Lancet,* vol. 353, pp. 2035-2036, Jun 12, 1999.

[20] S. J. Page, P. Levine and A. Leonard, "Mental practice in chronic stroke: results of a randomized, placebo-controlled trial,"  *Stroke,* vol. 38, pp. 1293-1297, Apr, 2007.

[21] C. Schuster, R. Hilfiker, O. Amft, A. Scheidhauer, B. Andrews, J. Butler, U. Kischka and T. Ettlin, "Best practice for motor imagery: a systematic literature review on motor imagery training elements in five different disciplines,"  *BMC Med.,* vol. 9, pp. 75-7015-9-75, Jun 17, 2011.

[22] A. Saimpont, M. F. Lafleur, F. Malouin, C. L. Richards, J. Doyon and H. P. Jackson, "The comparison between motor imagery and verbal rehearsal on the learning of sequential movements,"  *Front.  Hum.  Neurosci.,* vol. 7, pp. 773, Nov 18, 2013.

[23] D. L. Feltz and D. M. Landers, "The effects of mental practice on motor skill learning and performance: a meta-analysis,"  *J.   Sport Psych,* vol. 5, pp. 25-57, 1983.

[24] M. Lotze and U. Halsband, "Motor imagery,"  *J.  Physiol.  Paris,* vol. 99, pp. 386-395, Jun, 2006.

[25] G. Howatson, T. Zult, J. P. Farthing, I. Zijdewind and T. Hortobagyi, "Mirror training to augment cross-education during resistance training: a hypothesis,"  *Front.  Hum.  Neurosci.,* vol. 7, pp. 396, Jul 24, 2013.

[26] T. Zult, G. Howatson, E. E. Kadar, J. P. Farthing and T. Hortobagyi, "Role of the Mirror-Neuron System in Cross-Education,"  *Sports Med.,* Oct 13, 2013.

[27] J. P. Farthing, J. R. Krentz, C. R. Magnus, T. S. Barss, J. L. Lanovaz, J. Cummine, C. Esopenko, G. E. Sarty and R. Borowsky, "Changes in functional magnetic resonance imaging cortical activation with cross education to an immobilized limb,"  *Med.  Sci.  Sports Exerc.,* vol. 43, pp. 1394-1405, Aug, 2011.

[28] K. Amemiya, T. Ishizu, T. Ayabe and S. Kojima, "Effects of motor imagery on intermanual transfer: a near-infrared spectroscopy and behavioural study,"  *Brain Res.,* vol. 1343, pp. 93-103, Jul 9, 2010.

[29] M. I. Garry, A. Loftus and J. J. Summers, "Mirror, mirror on the wall: viewing a mirror reflection of unilateral hand movements facilitates ipsilateral M1 excitability,"  *Exp.  Brain Res.,* vol. 163, pp. 118-122, May, 2005.

[30] J. Decety, "The neurophysiological basis of motor imagery,"  *Behav.  Brain Res.,* vol. 77, pp. 45-52, May, 1996.

[31] R. C. Oldfield, "The assessment and analysis of handedness: the Edinburgh inventory,"  *Neuropsychologia,* vol. 9, pp. 97-113, Mar, 1971.

[32] A. van Lunteren, G. H. van Lunteren-Gerritsen, H. G. Stassen and M. J. Zuithoff, "A field evaluation of arm prostheses for unilateral amputees,"  *Prosthet.  Orthot.  Int.,* vol. 7, pp. 141-151, Dec, 1983.

[33] M. Lee and T. J. Carroll, "Cross education: possible mechanisms for the contralateral effects of unilateral resistance training,"  *Sports Med.,* vol. 37, pp. 1-14, 2007.

[34] E. A. Christou and T. M. Rodriguez, "Time but not force is transferred between ipsilateral upper and lower limbs,"  *J.  Mot.  Behav.,* vol. 40, pp. 186-189, May, 2008.

[35] D. Bensmail, A. S. Sarfeld, G. R. Fink and D. A. Nowak, "Intermanual transfer of sensorimotor memory for grip force when lifting objects: the role of wrist angulation,"  *Clin.  Neurophysiol.,* vol. 121, pp. 402-407, Mar, 2010.

[36] E. C. Chang, J. R. Flanagan and M. A. Goodale, "The intermanual transfer of anticipatory force control in precision grip lifting is not influenced by the perception of weight,"  *Exp.  Brain Res.,* vol. 185, pp. 319-329, Feb, 2008.

[37] L. A. Teixeira, "Timing and force components in bilateral transfer of learning,"  *Brain Cogn.,* vol. 44, pp. 455-469, Dec, 2000.

[38] J. H. Park and C. H. Shea, "Effector independence,"  *J.  Mot.  Behav.,* vol. 34, pp. 253-270, Sep, 2002.

[39] L. M. Parsons, "Imagined spatial transformations of one's hands and feet,"  *Cogn.  Psychol.,* vol. 19, pp. 178-241, Apr, 1987.
